# Gaze and Eye Tracking: Techniques and Applications in ADAS

**DOI:** 10.3390/s19245540

**Published:** 2019-12-14

**Authors:** Muhammad Qasim Khan, Sukhan Lee

**Affiliations:** Department of Electrical and Computer Engineering, Intelligent Systems Research Institute, Sungkyunkwan University, Suwon 440-746, Korea; qasim@skku.edu

**Keywords:** advanced driving assistance systems (ADAS), eye tracking, gaze tracking, line of sight (LoS), point of regard (PoR), road safety

## Abstract

Tracking drivers’ eyes and gazes is a topic of great interest in the research of advanced driving assistance systems (ADAS). It is especially a matter of serious discussion among the road safety researchers’ community, as visual distraction is considered among the major causes of road accidents. In this paper, techniques for eye and gaze tracking are first comprehensively reviewed while discussing their major categories. The advantages and limitations of each category are explained with respect to their requirements and practical uses. In another section of the paper, the applications of eyes and gaze tracking systems in ADAS are discussed. The process of acquisition of driver’s eyes and gaze data and the algorithms used to process this data are explained. It is explained how the data related to a driver’s eyes and gaze can be used in ADAS to reduce the losses associated with road accidents occurring due to visual distraction of the driver. A discussion on the required features of current and future eye and gaze trackers is also presented.

## 1. Introduction

### 1.1. Background and Motivation

The human eyes, a beautiful and interactive organ in the human body, have unique physical, photometric, and motion characteristics. These characteristics provide vital information required for eye detection and tracking. In our daily lives, a person’s emotional state, mental occupancy, and needs can be judged by the person’s eyes movements. Through our eyes, we identify the properties of the visual world and collect the information essential to our lives. Moreover, in the field of image and video processing, eyes play a vital role in the process of face detection and recognition [1,2,3,4]. The history of eye tracking dates back to second half of 18th century when researchers observed eye movements to analyze reading patterns. The early trackers used a sort of contact lens with a hole for the pupil [5]. In this arrangement, the movements of eye were tracked using an aluminum pointer connected to the lens. The authors of [6,7] developed first non-intrusive eye-trackers using light beams that were reflected on the eye and then recorded on a film. The authors also provided a systematic analysis of reading and picture viewing. A significant contribution in eye tracking research was made by the author of [8] in the 1950s and 1960s. The author showed that the gaze trajectories depend on the task that the observer has to execute. If the observers are asked particular questions about an image, their eyes concentrate on question-relevant areas of the image. The author also devised a suction cup that could stay on the human eye by suction to analyze visual perceptions in the absence of eye movements. In 1970s and afterwards, the research of eye tracking expanded rapidly [9]. In 1980s, a hypothesis known as the eye-mind hypothesis was formulated and critically analyzed by other researchers [10,11,12]. The hypothesis proposed that there is no considerable lag between what is fixated and what is processed. Further, several aspects related to eye tracking in the field of human-computer interaction and eye tracking applications to assist disabled people were also developed in the same decade [13]. During the last two to three decades, a revolutionary development was observed in eye tracking due to introduction of artificial intelligence techniques and portable electronics and head-mounted eye trackers.

**Figure 1:**
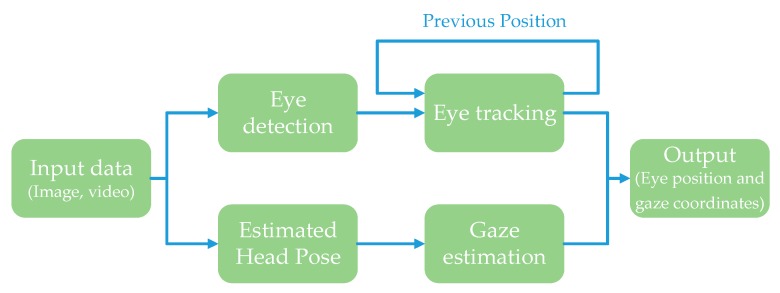
The process of tracking eye position and gaze coordinates.

Eye tracking and gaze estimation are essentially two areas of research. The process of eye tracking involves three main steps; viz., to discover the presence of eyes, a precise interpretation of eye positions, and frame to frame tracking of detected eyes. The position of the eye is generally measured with the help of the pupil or iris center [14]. Gaze estimation is a process to estimate and track the 3D line of sight of a person, or simply, where a person is looking. The device or apparatus used to track gaze by analyzing eye movements is called a gaze tracker. A gaze tracker performs two main tasks simultaneously: localization of the eye position in the video or images, and tracking its motion to determine the gaze direction [15,16]. A generic representation of such techniques is shown in Figure 1. In addition to its application in advanced driving assistance systems (ADAS), gaze tracking is also critical in several other applications, such gaze-dependent graphical displays, gaze-based user interface, investigations of human cognitive states, and human attention studies [17,18,19].

Tracking of driver’s eyes and gaze is an interesting feature of advanced driving assistance systems (ADAS) that can help reduce the losses involved in road accidents. According to World Health Organization’s reports [20,21,22], every year approximately 1–1.25 million people die and 20–50 million people receive injuries due to road accidents across the world. Moreover, if the recent trend of road accidents persists by 2030, road accidents could be the 5th main cause of death. In terms of cost, the damages involved in road accidents are more than five hundred billion USD. This amount is approximately equal to 2% of the gross national product (GNP) of advanced countries, 1.5% of the GNP of medium-income economies, and 1% of GNP of low-income countries. According to the recent studies (e.g., [23]), it is hoped that the amount of road accidents (related to visual distraction) will be reduced by 10–20% due to facial monitoring feature of ADAS.

### 1.2. Contribution and Organization

The intention of this paper is to benefit researchers by offering a comprehensive framework for a basic understanding of eye and gaze tracking and their applications in ADAS. To the best of authors’ knowledge, this is the first study that reviews the visual data (i.e., eyes and gaze data) techniques in the context of ADAS applications, though studies do exist regarding individual topics covered in this paper. 

This paper is organized as follows: Section 2 and Section 3 explain the models and techniques developed for eye and gaze tracking, respectively. The major categories of these models and techniques, with emphasis on the literature in which these techniques were initially proposed, and their respective benefits and limitations, are also discussed. Section 4 explains the driving process and challenges associated with a driver’s visual distraction. In this section, it is explained that how visual data of a driver are collected, processed, and used in ADAS applications. Further, the features of modern vehicles based on utilization of visual data of drivers and other vehicle parameters are summarized in this section. At the end of each section of the paper, necessary information is presented in a comprehensive tabular form. Section 5 concludes the paper with pointers on the future directions in this research field. The authors do admit that the topic presented is too wide and deep to be reviewed by a single paper. We encourage the interested readers to refer to other references, provided at the end of this paper, for further study of specific areas or the topics not covered in this work. For example, operational definitions of driving performance measures and statistics are well-documented in [24]. 

## 2. Eye Tracking

### 2.1. Introduction

**Figure 2:**
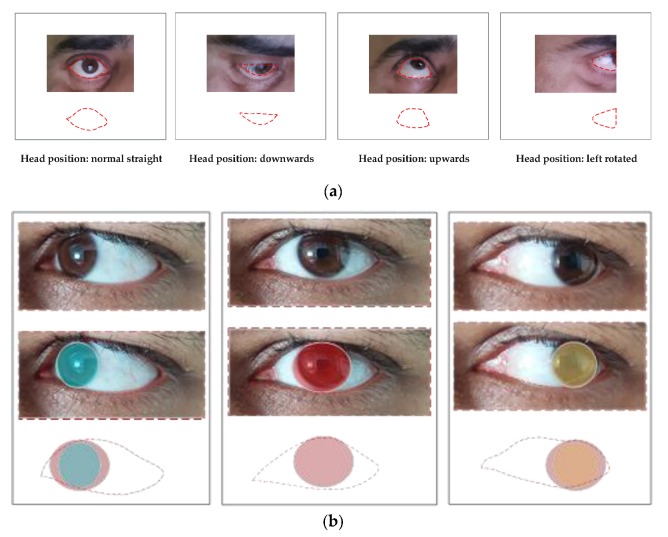
The appearances of eyes and eye parts change with head and eye movements. (**a**) Variability in eye appearance when eye position is fixed but head position varies. (**b**) Variability in gaze direction when head position is fixed but eyeball rotates.

The first step in eye tracking is to detect the eyes. The detection of eyes in image or video data is based on eye models. An exemplar eye model should be sufficiently meaningful to accommodate the variability in eyes’ dynamics and appearance while adequately constrained to be computationally efficient. Eye detection and tracking is an arduous job due to exceptional issues, such as degrees of eye openness; variability in size, head pose, and reflectivity; and occlusion of the eye by eyelids [3,25,26]. For instance, a small variation in viewing angle or head position causes significant changes in the eye appearance or gaze direction, as shown in Figure 2. The eye’s appearance is also influenced by ethnicity of the subject, light conditions, texture, iris position within eye socket, and the eye status (open or closed). Eye detection methods are broadly categorized based on eyes’ shape, features, and appearance, as explained below.

### 2.2. Shape-Based Techniques

An open eye can be efficiently expressed by its exterior (e.g., eyelids) and interior (e.g., iris and pupil) parts. The shape-based techniques are based on a geometric eye model (i.e., an elliptical or a complex eye structure) augmented with a similarity index. The model defines the allowable template deformations and contains parameters for nonrigid template deformations and rigid transformations. The main feature of these techniques is their capability of handling the changes in shape and scale.

#### 2.2.1. Elliptical Eye Models

For simpler applications of eye detection and tracking, the elliptical appearance of the eye can serve the job. Though simple elliptical eye shape models proficiently model features such as the pupil and iris under various viewing angles, these models are lacking in terms of capturing the variations and inter-variations of certain eye features. A major category of the techniques which consider the simple elliptical eye model are known as model fitting techniques which fit the designated features to the elliptical model [27,28]. Typically, in the techniques which utilize the elliptical eye model, pupil boundaries are extracted with the help of edge detection techniques, while transformation algorithms such as the Hough transform are utilized to extract the features of iris and pupil [29]. The authors of [30] and [31] estimated the center of pupil ellipse using thresholds of the image intensities. In their techniques, a constraint of shape circularity is employed to improve the efficiency; however, the model works only for near-frontal faces due to this constraint. Another category of techniques that exploit the simple elliptical eye model calls its members voting-based techniques [31]. The parameter selected in voting techniques support a given hypothesis through an accumulation process. The authors of [32] proposed a voting scheme that utilized temporal and spatial data to detect the eyes. They used a large temporal support and a set of heuristic rules to reject false pupil candidates. A similar voting scheme, which used edge orientation directly in the voting process, was also suggested in [33]. This technique was based on the intensity features of the images, and it relied on anthropomorphic averages and a prior face model to filter out the false positives. A limitation of such techniques is that they basically rely on maxima in feature space. When the number of eye region features decreases, the techniques may mistake other regions, such as eyebrows, for the eyes. So, these techniques are typically applicable when the search region is confined. A low-cost eye tracking system is proposed in [34], where the Starburst algorithm is used for iris detection. This algorithm finds the highest gray-level differences along rays while recursively sparkling new rays at the already found maxima. The Starburst algorithm is basically an active shape model which uses several features along each normal. 

#### 2.2.2. Complex Shape Models

Complex shape-based models are based on in-depth modeling of the eye shape [35,36,37,38]. A well-known example of complex shape models is the deformable template model [35], which consists of a circle for the iris representation and two parabolas for the eyelids. To fit the model to an image, energy functions for internal forces, edges, valleys, and image peaks, are incorporated in an update rule. However, the right selection of the template’s initial position is crucial for accurate results in this approach as the system cannot detect the eyes if the template is initialized above the eyebrow. Other limitations of this model are the complex template description and complexity with eye occlusions due to non-frontal head pose or eyelid closure. The authors of [36] extended this model to extract the eye features by considering eye corners as the initialization points. They used a nonparametric technique (known as snake model) to determine the head’s outline, and found the approximated eye positions by anthropomorphic averages. The information of the detected eye corners is utilized to lower the iterations number in the optimization of the deformable template. Similarly, the authors of [39,40] proposed the ways to speed up the technique proposed in [35]. Some researchers combined the features of complex eye models with elliptical models to improve accuracy and speed of the localization process (e.g., [41]).

Certain deformable models (e.g., snake model) can accommodate for significant shape variations, while the others cannot handle the large variability of eye shapes. The techniques based on deformable eye template are typically considered more logical, generic, and accurate. However, they have certain limitations, such as the requirement for high contrast images, being computationally demanding, and requiring initialization close to the eye. Moreover, for larger head movements, they subsequently rely on other techniques to provide good results.

### 2.3. Feature-Based Techniques

Feature-based techniques are based on the identification and utilization of a set of unique features of the human eyes. These techniques identify such local features of the eye and the face which have reduced sensitivity to variations in viewing angles and illumination. The commonly used features for eye localization are corneal reflections, limbus, and dark and bright pupil images. Typically, these techniques first identify and detect the local features; then, they apply a filter to highlight desired features while suppressing the others or utilize a prior eye shape model to construct a local contour; and, finally, they apply the classification algorithms to produce the output. Generally, the feature-based techniques are reported to provide good results in indoors applications; however, their outdoor performance is comparatively limited. These techniques are further subcategorized as follows.

#### 2.3.1. Local Features

Eyes’ local features are detected and utilized in combination with a prior shape model to detect and track the eyes [42,43,44,45,46]. For instance, the approach proposed in [42] first located a specific edge and then employed steerable Gabor filters to trail the edges of the eye corners or the iris. Next, based on the selected features and the eye model, a search policy was adopted to detect the shape, position, and corners of the eye.

The authors of [44] suggested a part-based model, in which an eye part (e.g., eyelid) is considered as a microstructure. They extracted face features using a multilayer perception method by locating eyes on face images. The authors of [45] extended the work of [42] and made improvements by utilizing multiple specialized neural networks (NN) trained to detect scaled or rotated eye images, and they worked effectively under various illumination conditions. The authors of [46,47] detected and utilized the information of area between the two eyes instead of eyes themselves. The area between the eyes is comparably bright on lower and upper sides (nose bridge and forehead, respectively) and has dark regions on its right and left sides. This area is supposed to be more stable and detectable than the eyes themselves. Moreover, this area can be viewed from a wide range of angles, and has a common pattern for most people. The authors of [46,47] located the candidate points by employing a circle-frequency filter. Subsequently, by analyzing the pattern of intensity distribution around the point, they eliminated the spurious points. Enhancing the robustness of this method, a fixed “between the eyes” template was developed to identify the actual candidates and to avoid the confusion between the eye regions and other parts [48,49]. 

#### 2.3.2. Filter Response

Use of specific filter was also proposed in several techniques to enhance a desired set of features while diminishing the impact of irrelevant features. For instance, authors of [50,51] used linear and nonlinear filters for eye detection and face modeling. They used Gabor wavelets for detection of edges of the eye’s sclera. The eye corners, detected through nonlinear filter, are utilized to determine the eye regions after elimination of the spurious eye corner candidates. The edges of the iris are located through a voting method. Experimental results demonstrate that the nonlinear filtering techniques are superior to the traditional, edge-based, linear filtering techniques in terms of detection rates. However, the nonlinear techniques require high-quality images. 

#### 2.3.3. Detection of Iris and Pupil

The pupil and iris being darker than their surroundings are commonly considered reliable features for eye detection. The authors of [52] used a skin-color model and introduced an algorithm to locate the pupils by searching for two dark areas that fulfill specific anthropometric requirements. Their technique, however, cannot perform well in different light conditions due to limitation of the skin-color model. Generally, use of IR light instead of visible light seems more appropriate for dark region detection. The techniques based on iris and pupil detection require the images taken from close to the eyes or high-resolution images.

The majority of the feature-based techniques cannot be used to model closed eyes. In an effort to overcome this limitation, a method [53] was proposed to track the eyes and to retrieve the eye parameters with the help of a dual-state (i.e., open or closed) eye model. The eyelids and eyes’ inner corners are detected through the algorithm proposed in [54]. This technique, however, requires a manual initialization of the eye model and high contrast images. 

### 2.4. Appearance-Based Techniques

The appearance-based techniques detect and track the eyes by using photometric appearance of the eyes, which is characterized by the filter response or color distribution of the eyes with respect to their surroundings. These techniques can be applied either in a spatial or a transformed domain which diminishes the effect of light variations. 

Appearance-based techniques are either image template-based or holistic in approach. In the former approach, both the intensity and spatial information of each pixel is maintained, while in the latter technique, intensity distribution is considered and the spatial information is disregarded. Image template-based techniques have limitations associated with scale and rotational modifications, and are negatively influenced by eye movements and head pose variations for the same subject. Holistic approaches (e.g., [55,56]) make use of statistical techniques to derive an efficient representation while analyzing the intensity distribution of the entire object’s appearance. The representation of the object, defined in a latent space, is utilized to deal with the disparities in the object’s appearance. During the test stages of the technique, the similarity analysis between the stored patterns and the test image is performed in the latent space. These techniques usually need a large amount of training data (e.g., the eyes of different subjects under different illumination conditions and facial orientations). However, the underlying developed models, constructed through regression, are principally independent of the object classes. 

### 2.5. Hybrid Models and Other Techniques 

Some techniques are based on symmetry operators [57,58,59] while some approaches exploit the data of eye blinks and motions [48,53,60,61,62]. Hybrid models combine the benefits of various eye models in a single arrangement while overcoming their deficiencies. These models, for instance, combine shape and intensity features [63,64,65], and shape and color features [52,62,63,64,65,66,67,68,69,70,71].

### 2.6. Discussion

The eye detection and tracking techniques, based on their photometric and geometric properties, are discussed in the preceding sections. Each technique has its own pros and cons, and the best performance of any scheme requires fulfillment of specific conditions in image and video data. These conditions are related to ethnicity, head pose, illumination, and degree of eye openness. The existing approaches are usually well applicable to fully open eyes, near-frontal viewing angles, and under good illumination conditions. Table 1 summarizes the various eye detection techniques and compares them under various image conditions.

## 3. Gaze Tracking

### 3.1. Introduction

**Figure 3:**
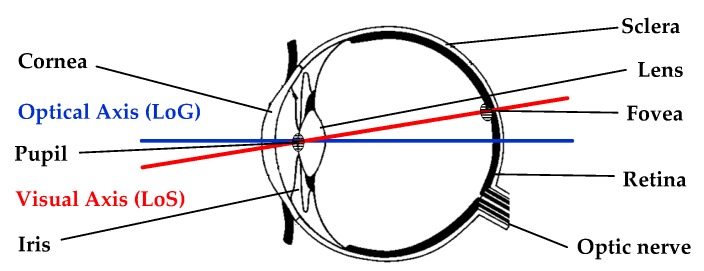
Structure of human eye.

The typical eye structure used in gaze tracking applications is demonstrated in Figure 3. The modeling of gaze direction is based either on the visual axis or on the optical axis. The visual axis, which forms the line of sight (LoS) and is considered the actual direction of gaze, is the line connecting the center of the cornea and the fovea. The optical axis, or the line of gaze (LoG), is the line passing through the centers of pupil, cornea, and the eyeball. The center of cornea is known as the nodal point of the eye. The visual and optical axes intersect at the nodal point of the eye with a certain angular offset. The position of head in 3D space can be directly estimated by knowing the 3D location of the corneal or eyeball center. In this way, there remains no need for separate head location models. Thus, the knowledge of these points is the keystone for majority of the head pose invariant models [87,88].

The objective of gaze tracking process is to identify and track the observer’s point of regard (PoR) or gaze direction. For this purpose, the important features of eye movements such as fixation, saccades, and smooth pursuit are utilized. Fixation represents the state when the observer’s gaze rests for a minimum time (typically more than 80–100 ms) on a specific area within 2–5° of central vision. Saccades are quick movements of eyes that take place when visual attention transfers between two fixated areas, with the aim of an bringing area of interest within the narrow visual field. When a driver visually follows a traveling object, this state is represented by smooth pursuit [62]. The data associated with the fixations and saccades provides valuable information that is used for the identification and classification of vision, neurological, and sleep conditions. In the field of medical psychology, data of the fixations is utilized to analyze a person’s attentiveness and level of concentration. Saccadic eye movements are widely studied in a variety of applications such as human vision research and drowsiness detection for vehicle drivers. Moreover, saccade is also used as a helpful index for determination of mental workload. Studies show that the saccade distance decreases when the task’s complexity increases [89].

The gaze tracking systems take two parameters as the input: eyeball orientation and head pose (defined by the orientation and position of the head) [90]. To change the gaze, a person can move his (or her) head while keeping the position of eyes fixed with respect to the head. Alternatively, gaze direction can also be changed by moving the eyeballs and pupil while the head is at rest. These two practices are respectively named “owl” and “lizard” vision in [91] because of their resemblance with these animals’ viewing behavior. Normally, we first move our heads to a comfortable position and then orient our eyes to see something. In this process, the head pose defines the gaze direction on a coarse scale, whereas the fine scale gaze direction is determined by the eyeball orientation. More specifically, to further understand the correlation between the head pose and eye pose, the study in [91] investigates two question: (i) How much better can gaze classification methods classify driver gaze using head and eye pose versus using head pose only? (ii) With the addition of eye pose information, how much does gaze classification improve? Generally, information of both the head pose and the pupil position is required in gaze estimation applications. As it will be explained in the later sections, the information of head pose is usually incorporated implicitly in gaze estimation applications rather than directly. An important aspect of gaze tracking process is the head pose invariance. The resultant head position invariance is achieved with the help of specific configurations of multiple cameras and other sensors whose a priori knowledge is available in the algorithms. 

There are various configurations of lights and cameras, such as single camera, single light [88,92,93,94]; single camera, multiple lights [85,95,96,97,98]; and multiple cameras, multiple lights [30,99,100,101,102,103]. A complementary practice performed in all gaze tracking schemes is known as calibration. During the calibration process, elements of gaze tracking system are calibrated to determine a set of useful parameters, as explained below.

1. Calibration of geometric configuration of the setup is necessary to determine the relative orientations and locations of various devices (e.g., light sources and cameras).
2. Calibration associated with individuals is carried out to estimate corneal curvature—the angular offset between optical and visual axes.
3. Calibration of eye-gaze mapping functions according to the applied method.
4. Calibration of the camera is performed to incorporate the inherent parameters of the camera.

Certain parameters such as human specific measurements are calculated only once, whereas the other parameters are determined for every session by making the subject gaze at a set of specific points on a display. The parameters associated with devices, such as physical and geometric parameters of angles and locations between various devices, are calibrated prior to use. A system is considered fully calibrated if the geometric configuration and camera parameters are accurately known. 

After introducing the basic concepts related to gaze tracking, the major techniques of gaze tracking are explained as follows.

### 3.2. Feature-Based Techniques

Feature-based gaze tracking techniques use eyes’ local features for gaze estimation. These techniques are broadly categorized as the model-based and the interpolation-based techniques, as explained below. 

#### 3.2.1. Model-Based Techniques

The model-based techniques use the geometric model features of the eye to directly calculate the gaze direction. The point of gaze is determined by the intersection of the gaze path with the object of a gaze [90,97,99,101,104]. These techniques model the general physical structure of the eye in geometric forms to estimate a 3D gaze direction vector. The PoR is calculated as the intersection of the closest object in the scene with the gaze direction vector. 

Typically, there are three categories (i.e., intrinsic, extrinsic, and variable) of the parameters utilized for development of the geometric model of the eye [99]. The intrinsic parameters, calculated for fixed eye, remain unchanged during a tracking session; however, they change gradually over the years. These parameters include iris radius, cornea radius, the distance between the centers of the cornea and the pupil, the angle between optical and visual axes, and refraction parameters. The extrinsic parameters such as pupil radius are used to develop a model for optical axis and 3D eye position. These models adjust the shape of the eye according to the parameters. 

Most 3D model-based techniques (e.g., [88,90,96,97,102,104,105,106,107,108] depend on metric information, and consequently, call for a global geometric model of orientation and position of devices and light sources. Further, camera calibration is also critical in these techniques. Some exceptional approaches use simplified assumptions [27] or use projective invariants [95,98]. We will not discuss the mathematical details of these techniques; however, most of them work on the same fundamental principles. The calibrated output of cameras is utilized to measure the lengths and angles by applying Euclidean relations. The general strategy is to make an assessment of the center of the cornea, and then to develop a model of optical axis. The points on the visual axis cannot be measured directly from the images. However, the offset to the visual axis is estimated by showing one or more points on the screen. The intersection of the visual axis and the screen in a fully calibrated setup provides the PoR. 

In a model-based technique, the corneal center, which is the point of intersection of visual and optical axes, is considered as an important parameter for gaze estimation. If the corneal curvature is already known, it is possible to determine the corneal center with the help of two light sources and a camera. For estimation of corneal curvature, anthropomorphic averages are usually adopted due to their simplicity and ease of use [107,109]. However, if the eye-related parameters are unidentified, at least two cameras and two light sources are required to estimate the corneal center [96]. Several studies, such as [88,102,110], used model-based techniques in a fully calibrated arrangement. At a minimum, a single point of calibration is mandatory to estimate the angle between the visual and optical axes. This angle is used to estimate the direction of gaze [102]. 

#### 3.2.2. Interpolation-Based Techniques 

The regression-based methods (e.g., [27,69,100,111,112,113,114,115]), on the other hand, map the image features to the gaze coordinates. They either have a nonparametric form, such as in neural networks [113,116] or a specific parametric form, such as polynomials [112,117]. In initial gaze tracking applications, a single source of IR light was employed to enhance the contrast and consequently produce stable gaze estimates. Many single-glint techniques were implicitly based on an erroneous assumption that “the corneal surface is a perfect mirror.” This assumption inferred that the glint should remain stationary as long as the head position is fixed even when the corneal surface rotates. Therefore, the glint is taken as the origin in glint-centered coordinate systems. In this view, the difference between the pupil center and the glint is utilized to estimate the gaze direction. So, the pupil-glint difference vector is typically mapped to the screen. The authors of [118] developed a video-based eye tracker for real-time application. They used a single camera and employed IR light for dark bright pupil images. To compensate for head movements, they considered a set of mirrors and galvanometers. The PoR was estimated by using a linear mapping and the pupil-glint vector. The higher values of pupil-glint angles were considered to correspond to nonlinearities. They used polynomial regression to compensate for these nonlinearities. Similarly, in a later study, the authors of [73] represented a mapping of glint-pupil difference vector to the PoR. They utilized a single camera and considered a 2nd order polynomial to calculate the x and y-coordinates. However, as explained in [112], as the head moves farther from its initial position, decay in the calibration mapping is observed. In a way similar to [73] and [118], the authors of [119] proposed a polynomial regression technique for estimation of the PoR while assuming a flat cornea surface. Additionally, to compensate the gaze imprecision due to lateral head movements, they proposed a first order linear interpolation model. The results of these studies suggest that the higher order polynomials do not deliver superior calibration in practical applications. The findings of [119] are also supported by the results of [88,96]. 

For interpolation tasks, NNs and their variants are frequently adopted. The authors of [120] suggested a generalized NN-based regression technique in which the glint coordinates, pupil-glint displacement, pupil parameters, and ratio and orientation of the pupil ellipse’s major and minor axes are utilized to map the screen coordinates. The main objective of this technique is to eliminate the need for calibration after having performed the initial training. The results of the technique are accurate within 5° even in the presence of head movements. In [121], the authors used support vector regression to construct a highly non-linear generalized gaze mapping function that accounts for head movement. The results of this technique show that eye gaze can be accurately estimated for multiple users under natural head movement. Most gaze tracking techniques are unable to distinguish if the present input (or test data) is no longer compatible with the training or calibration data. So, the authors of [69,116] used the covariance of the test and training data to indicate when the gaze estimates significantly diverge from the training data. 

It is observed that the head pose changes are not properly addressed by 2D interpolation techniques even with the eye trackers mounted on the head as these trackers might slip and change their position. To adjust minor slippage of head mounts, the authors of [122] proposed a set of heuristic rules. The single camera based 2D interpolation techniques indirectly model the eye physiology, geometry, and optical properties; and are typically considered approximate models. It is notable that head pose invariance is not strictly guaranteed in these models. However, their implementation is simple without requiring geometric or camera calibration, and they produce reasonably acceptable results for minor head movements. Some interpolation-based techniques try to improve the accuracy under increased head movements by using additional cameras, or through compensation [123]. The authors of [123] introduced a 2D interpolation-based technique to estimate 3D head position with the help of two cameras. They modified the regression function using the 3D eye position to compensate for head motions. However, in contrast to other interpolation-based techniques, the technique in [123] requires a prior calibration of the cameras. 

### 3.3. Other Techniques

Most gaze estimation techniques are based on feature extraction and use IR light. However, in the following subsections, some alternative approaches are discussed which are based on different lines of action. These techniques utilize the reflections from the eye layers (Purkinje image), in contrast to of extracting iris and pupil features [124,125], appearance-based techniques [89,114,126], and the techniques that use visible light [114,116,127].

#### 3.3.1. Appearance-Based Techniques

The appearance-based gaze estimation techniques take the contents of an image as input with the objective of mapping them directly to PoR on the screen. Accordingly, the underlying function to estimate the personal variations has relevant features extracted implicitly, without requiring the calibration of cameras and geometry. These techniques employ cropped images of the eye for training of the regression functions as observed in Gaussian process [114], multilayered networks [68,89], and manifold learning [128]. The authors of [114] obtained gaze predictions and related error measurements by using a sparse Gaussian process interpolation technique on filtered images in visible spectrum. The technique in [128] learned the eye image manifold by employing locally linear embedding. This technique significantly reduces the number of calibration points without sacrificing the accuracy. The accuracy of results of [128] is comparable to that of [89]. 

Appearance-based techniques normally do not necessitate the camera and geometric calibration as the mapping is performed directly on the contents of the images. While appearance-based techniques aim to model the geometry in an implicit manner, head pose invariance has not been reported in the literature. Moreover, since a change in illumination may alter the eye appearance, the accuracy of these techniques is also affected by the different light conditions for the same pose.

#### 3.3.2. Visible Light-Based Techniques

The techniques based on visible or natural light are considered a substitute for the techniques based on IR, especially for outdoor daylight applications [27,34,69,76,90,106,114]. However, they have limitations due to the light variations in the visible spectrum and poor contrast images. 

The authors of [76] modeled the visible part of the subject’s eyeball as a planar surface. They regarded gaze shifts due to eyeball rotations as translations of the pupil. Considering the 1-to-1 mapping of the projective plane and the hemisphere, the authors of [27] modeled the PoR as a homographic mapping to the monitor from center of the iris. The resultant model represents an approximation only as it does not consider the nonlinear one-to-one mapping. Moreover, this technique does not provide the head pose invariant models. The techniques developed in [90,106,127] estimated gaze direction by employing stereo and face models. The authors of [106] modeled the eyes as spheres and estimated the PoR from the intersection of the two estimates of LoG for each eye. In their work, a head pose model is used to estimate the eyeball center, and personal calibration is also considered. The authors of [90,127] combined a narrow-view-field camera with a face pose estimation system to compute the LoG through the one iris [90] and two irises [127], respectively. They assumed iris contours to be circles to approximate their normal directions in three dimensions by proposing novel eye models. Gaze estimation techniques that use rigid facial features are also reported in other studies, such as [63,129,130]. The locations of eye corners and the iris are tracked by means of a single camera, and the visual axis is estimated by employing various algorithms. The authors of [131] proposed the use of stereo cameras in natural light to estimate the gaze point. While these techniques do not require an IR light source, their accuracy is low as they are in the initial stages of development. 

Finally, it is notable that a lack of light at night time reduces the functionality of human vision and cameras, which results in increased pedestrian fatalities occurring at night. The authors of [132] proposed an approach which utilized cost-effective arrayed ultrasonic sensors to detect traffic participants in low-speed situations. The results show an overall detection accuracy of 86%, with correct detection rates of cyclists, pedestrians, and vehicles at around 76.7%, 85.7%, and 93.1%, respectively.

### 3.4. Discussion

The gaze tracking systems which present negligible intrusiveness and minimal usage difficulty are usually sought-after as they allow free head movements. In the modern gaze tracking applications, video-based gaze trackers are gaining increased popularity. They maintain good accuracy (0.5° or better) while providing the user with enhanced freedom of head movement. The recent studies indicate that high-accuracy trackers can be realized if some specific reflections from the cornea are utilized. Furthermore, the resultant gaze estimation is more stable and head pose invariant. However, unfortunately, commercially available, high-accuracy gaze trackers are very expensive. Moreover, there is a trade-off among accuracy, setup flexibility, and the cost for gaze trackers. The readers can find a thorough discussion on performance and preferences of eye tracking systems in [133]. A comprehensive comparison of gaze estimation methods is provided in Table 2. 

## 4. Applications in ADAS

### 4.1. Introduction

A driver’s gaze data can be used to characterize the changes in visual and cognitive demands to assess the driver’s alertness [136,137]. For instance, it is reported that increased cognitive demand impacts the drivers’ allocation of attention to the roadway [138,139,140,141]. With the increase of cognitive demand, drivers tend to concentrate their gaze in front of the vehicle. This gaze concentration results in a reduced frequency of viewing the speedometer and mirrors, and a reduced ability to detect in both peripheries [138,139,140,142,143,144,145]. These practices are consistent with unintentional blindness, loss of situational awareness, and situations such as ‘‘looked but failed to see’’ [139,143,146]. 

A prominent and intuitive measure to detect the changes in drivers’ gaze due to increased cognitive demand is percent road center (PRC). PRC is defined as “the percentage of fixations that fall within a predefined road center area during a specific period.” It has been shown that PRC increases with increased cognitive demand [136,141,142,143,147]. While the concept of PRC is simple to understand, the definition of road center differs significantly in the literature. It is defined either as a rectangular region centered in front of the vehicle having a width of 15° [141] and 20° [142], or a circular region of 16° diameter centered about the road center point [142] and centered on the driver’s most recurrent gaze angle [148]. Some implementations of PRC utilized raw gaze points and gaze trajectories recorded by eye trackers that were not clustered into saccades and fixations. The authors of [148] compared these approaches and observed a strong correlation between raw gaze-based PRC and fixation-based PRC. To characterize the variations in gaze behavior with cognitive demand, standard deviation of gaze points is also used by several researchers [137,138,139,142]. The standard deviation is either computed from the projection of the driver’s gaze trail on a plane or the driver’s gaze angle. A comparison of various techniques used to characterize the changes in drivers’ gaze under cognitive load is presented in [149].

**Figure 4:**
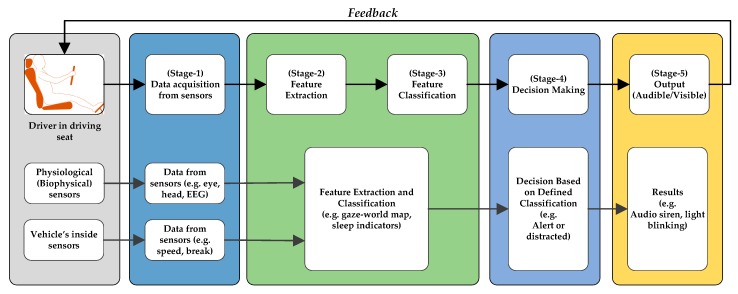
The stages of visual data in typical advanced driving assistance systems (ADAS) algorithms.

The data associated with driver’s eyes and gaze is utilized by the ADAS algorithms to detect the driver’s attentiveness. A typical scheme adopted in the ADAS algorithms to detect and improve the driver’s alertness using usual visual data of the driver is shown in Figure 4. These algorithms continuously capture the driver’s visual data through numerous sensors associated with the driver’s body and installed inside the vehicle. The obtained visual data is processed at the next stages to extract and classify the vital features. At the subsequent stage, a decision is made on the basis of data classification. The decision is conveyed to the driver in form of audible or visible signals, as shown in Figure 4. 

The subsequent sections present a detailed review of the systems and techniques that are used to detect the visual activities and distraction of a driver. A brief overview of driving process and associated challenges, however, seems feasible for better understanding of the subsequent sections.

### 4.2. Driving Process and Associated Challenges

**Figure 5:**
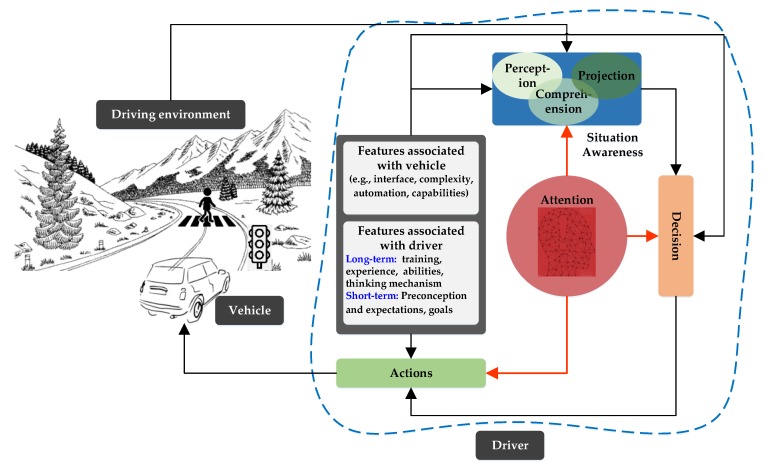
Driving process.

The key elements of the driving process are driver, vehicle, and driving environment, as shown in Figure 5. The driver, who plays the pivotal role in this process, has to understand the driving environment (e.g., nearby traffic and road signals), make decisions, and execute the appropriate actions [150]. Thus, the driver’s role has three stages: situational awareness, decision, and actions. Situational awareness is considered to be the most important and complicated stage which can be modeled as a three-step process. The first step is to perceive the elements in the environment within specific limits of time and space. The second step is to comprehend the relative significance of the perceived elements; and, the final step is to project their impact in near future. A driver’s ability to accurately perceive multiple events and entities in parallel depends on his (or her) attention during the first step (i.e., perception); and consequently, the situational awareness stage principally depends on it. Regarding the driver’s attention, is necessary to take in and process the available information during the decision and actions stages as well. Moreover, in a complex and vibrant driving environment, the need for the driver’s active attention increases, in order to save life and property. Thus, the ADAS continuously monitors the driver’s attention and generates an alarm or a countermeasure if any negligence is observed. The level of the alarm or countermeasure depends on the nature and intensity of the negligence. 

The recent studies [151,152] explain that there are three major causes of road accidents that contribute to more than 90% of total road accidents. These causes are: distraction, fatigue, and aggressive driver behavior. The term “fatigue” denotes a compromised mental or physical performance and a subjective feeling of drowsiness. For drivers, the most dangerous types of fatigue are mental and central nervous fatigues which ultimately lead to drowsiness. Other types of fatigue include local physical fatigue (e.g., skeletal muscle fatigue) and general physical fatigue which is typically felt after an exhaustive physical activity. Aggressive driving activities such as shortcut maneuvers and ignoring speed limits also constitute to major reasons for road accidents. Since they are primarily related to a driver’s intended actions, local traffic rules seem more effective than mere warnings from ADAS. Nevertheless, ADAS systems are capable of warning, and in near-autonomous vehicles, preventing the severe consequences. Distraction is defined as the engagement of a driver in a competitive parallel task other than driving [153]. 

The driver’s performance is severely affected by the distraction, and it is considered the main reason for nearly half of the total accidents [154,155]. There are several distracting activities, such as eating, drinking, texting, calling, using the in-vehicle-technology, and viewing at the off-road environment [156,157,158,159]. According to the NHTSA, these activities are categorized as [155,159,160]:

- Visual distraction (taking the eyes off the road);
- Physical distraction (e.g., hands off the steering wheel);
- Cognitive distraction (e.g., mind off the duty of driving);
- Auditory distraction (e.g., taking ears off of the auditory signals and honks).

### 4.3. Visual Distraction and Driving Performance

Human beings have limited capability to perform multiple tasks simultaneously without compromising the performance of the all tasks. Therefore, engaging in a competing task while driving degrades the driver’s performance; and, consequently, endangers traffic safety. Driving behavior can be evaluated with certain driving performance indicators [161,162]. These indicators include: lateral control, reaction time, and speed, as discussed below. 

#### 4.3.1. Lateral Control

Typically, the lateral control is affected by visual distraction. The distracted drivers ultimately maneuver larger deviations in lane positioning as they need to compensate for slip-ups made while their eyes were off the road. This increased lane-position variability has been reported by several researchers (e.g., [140,163]). Moreover, as reported in [140], the steering control of distracted drivers is less smooth in comparison to their attentive driving states. On the other hand, the author of [164] found that there is no significant difference in the standard deviation of lateral control for distracted and normal drivers. The difference in findings of the researchers could be due to different test conditions and varying driving behaviors. 

#### 4.3.2. Reaction Time

Reaction time is calculated by numerous measures, such as brake reaction time (BRT), detection response time (DRT), and peripheral detection time (PDT). These reaction times provide a measure of the driver’s mental load. Usually, the reaction time increases for visually distracted drivers [165,166,167].

#### 4.3.3. Speed

A driver’s distraction due to visual stimuli typically results in a speed reduction [147,163,168]. The reduced speed is perhaps the result of a compensatory mechanism for a potential risk as the potential risk can be minimized through a reduced speed. However, contradictory findings are reported in [164]. The authors of [164] observed an increased average speed and several speed violations for distracted drivers. The authors reasoned that the very low noise inside the vehicle was reason for the inconsistencies as the driver, thinking that the vehicle is at normal speed, did not monitor the speedometer frequently. We believe that since different researchers have different simulation or test environments (e.g., nearby vehicles, road conditions), differences between or opposition to each other’s findings are natural. Moreover, the behavior of different distracted drivers in respect to speed control is not always the same. 

### 4.4. Measurement Approaches

Researchers have exploited the features of eye movement data for driver’s distraction and drowsiness detection [169,170]. The following features related to eyeball and eyelid movements are frequently used in this field [171,172,173,174].

- *PERCLOS*: It is a measure of percentage of eye closure. It corresponds to the percentage of time during a one-minute period for which the eyes remain at least 70% or 80% closed.
- *Percentage eyes >70% closed (PERCLOS70).*
- *Percentage eyes >80% closed (PERCLOS80).*
- *PERCLOS70 baselined.*
- *PERCLOS80 baselined.*
- *Blink Amplitude*: Blink amplitude is the measure of electric voltage during a blink. Its typical value ranges from 100 to 400 μV.
- *Amplitude/velocity ratio (APVC).*
- *APCV with regression.*
- *Energy of blinking (EC).*
- *EC baselined.*
- *Blink Duration:* It is the total time from the start to the end of a blink. It is typically measured in the units of milliseconds. A challenge associated with blink behavior-based drowsiness detection techniques is the individually-dependent nature of the measure. For instance, some people blink more frequently in wakeful conditions or some persons’ eyes remain slightly open even in sleepy conditions. So, personal calibration is a prerequisite to apply these techniques.
- *Blink Frequency:* Blink frequency is the number of blinks per minute. An increased blink frequency is typically associated with the onset of sleep.
- *Lid Reopening Delay*: It is measure of the time from fully closed eyelids to the start of their reopening. Its value is in the range of few milliseconds for an awake person; it increases for a drowsy person; and is prolonged to several hundred milliseconds for a person undergoing a microsleep.
- *Microsleep:* An eye blink is detected when the upper lid of the eye remains in contact with the lower lid for around 200–400 ms, and if this duration exceed 500 ms (and less than 10 s), this situation corresponds to a microsleep [173,175]. A driver’s microsleep can lead to fatal accidents.
- *Microsleep event 0.5 sec rate.*
- *Microsleep event 1.0 sec rate.*
- Mean square eye closure.
- *Mean eye closure.*
- *Average eye closure speed.*

A driver’s physical activities such as head movements are captured and processed in the ADAS applications [176,177,178,179]. The video cameras are installed inside the vehicle at suitable locations to record the driver’s physical movements and gaze data. The main advantage of video-based gaze detection approaches lies with its nonintrusive nature [180,181,182,183]. For instance, the authors of [176] modeled and detected a driver’s visual distraction using the information associated with pose and position of the driver’s head. However, both intuitively and when explained by the authors, this technique is prone to report false positives. The primary reason for this is the possibility of the driver looking on the road while his (or her) head is tilted to a side. This study also explains the need for high-performance eye and gaze tracking systems for ADAS. The author of [177] proposed an improved technique by incorporating the PRC of gaze direction. They analyzed it over a 1 min epoch. For their setup, they found that PRC < 58% was a result of visual distraction, whereas PRC > 92% was due to cognitive distraction. 

The authors of [184] reported a correlation between driving performance and visual distraction by utilizing gaze duration as a detection feature. The existence of such correlation was also confirmed by the authors of [185]. It has been reported that the detection accuracy observed through using eye-movement data alone is nearly equal to that observed thorough using both eye-movement and driving performance data [185]. As reported in earlier studies and verified by recent research [186,187,188,189,190], eye-movement features can be effectively used for detection of visual as well as cognitive distraction. Distracted drivers are found to exhibit longer fixation durations or frequent fixations towards competing tasks. It is also observed that, a cognitively distracted driver usually exhibits longer fixation duration at the same area. The area of fixation can be either associated with a competing task (e.g., multimedia inside the vehicle) or with the peripheries of the field of view. 

The combined effect of visual and cognitive distraction is also reported in [140]. It is notable that, by definition, visual distraction is different from cognitive distraction (which includes the state “looked but did not see”), and their effects are also not the same. Cognitive distraction disturbs the longitudinal control of the vehicle, whereas visual distraction affects the vehicle’s lateral control and steering ability of a driver [191]. Moreover, as discussed in [140], overcompensation and steering neglect is related to the visual distraction, whereas under-compensation is associated with cognitive distraction. Similarly, hard braking is mostly related to the cognitive distraction [136,141]. Typically, the accidents due to visual distraction are more disastrous compared to the accidents because of cognitive distraction. The findings of [50] suggest that during visual distraction only the frequency and duration of eye fixations is higher than the combined (visual as well as cognitive) distraction. However, the frequency and duration of eye fixations during combined distraction is higher than that of cognitive distraction alone. It is notable that for adequate situation awareness there must be a specific range of suitable duration and frequency of eye fixation that depends on the driver and driving environment. Therefore, eye movement features can be helpful in order to accurately discriminate between visual and cognitive distraction only if the specific range of eye-movement features is pre-identified for each driver.

In addition to already explained physical measures, biological measures such as electrooculography (EOG) also provide data for sleepiness detection. EOG signals are frequently used to measure eye-related activities for medical purposes; however, their use in ADAS applications is accompanied with certain challenges. For example, the location of EOG electrodes has a special significance in its applications, as the accuracy of the collected data depends on distance of the electrodes from the eyes [192,193]. At the same time, it was observed that drivers do not feel comfortable with the electrodes attached to their eyes during normal driving situations. So, such experimentation is possible for simulation-based studies but not feasible for real-world applications. 

Realizing the relative advantages and limitations of the above-discussed techniques, the researchers now tend to fuse various techniques to produce an optimal solution for distraction detection systems of ADAS. By merging the information obtained from vehicle’s parameters (e.g., turning speed, and acceleration) and driver’s physical and biological parameters, more accurate and reliable results are reported. For example, the authors of [194] reported the distraction detection accuracy to be 81.1% by fusing the data of saccades, eye fixation, lateral control, and steering wheel through a support vector machine algorithm. The authors of [195] detected driver’s distraction by processing the information obtained from physical (blink frequency, location, and eye-fixation duration) and driving performance parameters (steering wheel and lateral control). Using the same physical parameters, the authors of [196] considered different driving performance measures (i.e., speed, lateral acceleration, and longitudinal deceleration) to detect the driver’s distraction. The authors of [197] merged biological and physical parameters (head orientation, gaze data, and pupil diameter) to produce more accurate results (91.7% and 93%) using support vector machine and adaptive boosting (Adaboost) algorithms, respectively. A summary of measurement techniques, their advantages, and their limitations are presented in Table 3.

### 4.5. Data Processing Algorithms

The data of driver’s eyes and gaze has information associated with the driver’s level of alertness. The following features of driver’s visual data are frequently used in ADAS applications:

- Difference between the maximum and minimum value of the data;
- Standard deviation of the data;
- Root mean square value of the data;
- Duration of the signal data;
- Maximum difference between any two consecutive values;
- Median of the data;
- Mean of the data;
- Maximum value of the data;
- Minimum value of the data;
- Amplitude of the difference between the first value and the last value;
- Difference between the max and min value of the differential of data.

**Figure 6:**
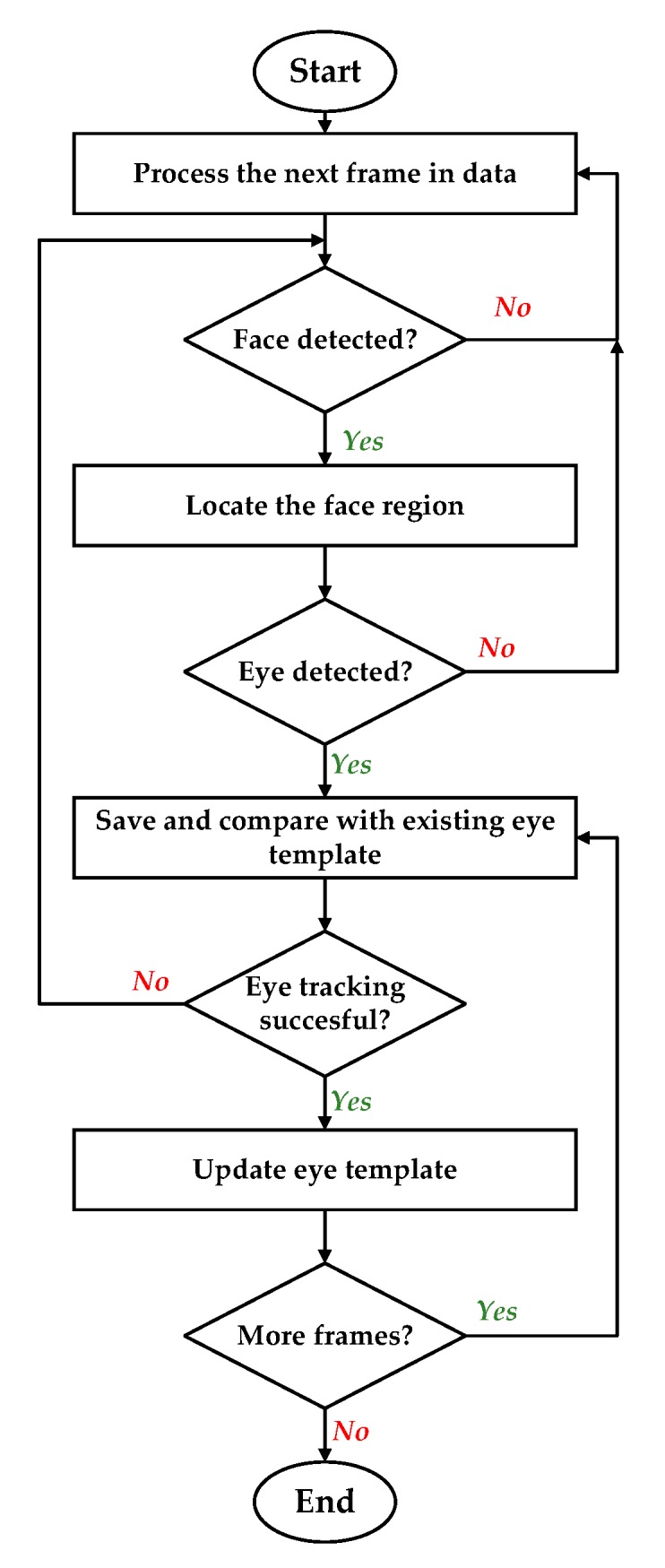
Flowchart of a generic eye tracking algorithm.

There are various algorithms developed and implemented by researchers to model and utilize eye and gaze data for detection of a driver’s alertness and intentions. These algorithms use fuzzy logic [198,199,200,201]; neural networks [202,203]; Bayesian networks [113,204,205]; unsupervised, semi-supervised, and supervised machine learning techniques [186,189,206]; and combinations of multiple techniques. It is logical that depending upon the usage and available resources, the processing algorithms select and process the data or part of it. For example, the authors of [207] argued that it is sufficient to partition gaze into regions for the purpose of keeping the driver safe. Their proposed approach, which estimates driver’s gaze region without using eye movements, extracts facial features and classifies their spatial configuration into six regions in real time. They evaluated the developed system on a dataset of 50 drivers from an on-road study while resulting in an average accuracy of 91.4% at an average decision rate of 11 Hz. Furthermore, algorithms for special circumstances such as during hazy weather are also discussed in the literature and belong to already discussed categories. For instance, the work in [208] is based on deep learning approaches. In general, all of these algorithms execute a recursive process similar to the flowchart shown in Figure 6. The presented flowchart shows, for example, how eye tracking is achieved in the ADAS applications. The main steps shown in the flowchart can be realized by application of any suitable conventional or modern algorithm. 

Additionally, the eye and gaze data are also used for early detection of a driver’s intentions, which is an interesting feature of ADAS. Most schemes developed for prediction of a driver’s maneuvering behavior are principally based on the hidden Markov model (HMM) and its variants [209,210,211,212]. These schemes are applied to the data obtained from the driver’s gaze sequence [9] and head position [213]. To process the data, feature-based pattern recognition and machine learning techniques are frequently utilized [214,215,216]. These schemes are designed to either detect a single maneuver behavior such as lane change only, or turn only [211,214,217,218,219] or multiple maneuver behaviors [220]. For instance, early detection of intention to change the lane was achieved in [221] using HMM-based steering behavior models. This work is also capable of differentiating between normal and emergency lane changes. Similarly, researchers utilized the relevance vector machine to predict driver intentions to change lanes [222], apply brakes [223], and take turns [224]. Moreover, by applying artificial neural network models on gaze behavior data, the authors of [202] conjectured the driver’s maneuvering intentions. In [206], deep learning approaches were utilized for early detection of the driver’s intentions. In this work, recurrent neural network (RNN) and long short-term memory (LSTM) units were combined which fuse the various features associated with the driver and the driving environment to predict the maneuvers. These features included the face and eye-related features captured by a face camera, and the driving parameters and street map and scene. The system developed in [206] can predict a maneuver 3.5 s earlier, together with the recall performance of 77.1% and 87.4% and the precision of 84.5% and 90.5% for an out of the box and a customized optimal face tracker, respectively. In addition to feature-based pattern recognition algorithms, linguistic-based syntactic pattern recognition algorithms are also proposed in the literature for early detection of driver’s intent [220]. The authors of [225] adopted the random forest algorithm and utilized the data of transition patterns between individual maneuver states to predict driving style. They showed that use of transition probabilities between maneuvers resulted in improved prediction of driving style in comparison to the traditional maneuver frequencies in behavioral analysis. Table 4 presents a summary of data processing algorithms used in ADAS that utilize a driver’s eye and gaze data for detection of distraction and fatigue.

### 4.6. Application in Modern Vehicles

Vehicle manufacturing companies use the features of drivers’ visual data to offer services and facilities in high-end models their vehicles. These vehicles are equipped with cameras, radars, and other sensors to assist drivers in safe and comfortable driving. For example, the Cadillac Super Cruise system utilizes FOVIO vision technology developed by Seeing Machines. In this system, a gumdrop-sized IR camera is installed on the steering wheel column to precisely determine the driver’s alertness level. This is achieved through an exact measurement of eyelid movements and head orientation under a full range of day and night-time driving conditions. The system is capable of working well even when the driver is wearing sunglasses. Table 5 summarizes the features offered by vehicle manufacturing companies.

## 5. Summary and Conclusions

This paper reviewed eye and gaze tracking systems—their models and techniques, the classification of techniques, and their advantages and shortcomings. Specifically, their application in ADAS for safe and comfortable driving has been discussed in detail. While these tracking systems and techniques show improvement in ADAS applications, there exists a significant potential for further developments, especially due to emergence of autonomous vehicle technology. The National Highway Traffic Safety Administration (NHTSA) of the USA defines six levels of vehicle automation to provide a common interface for research and discussions among different agencies, companies, and stakeholders [241]. These levels range from no automation (level-0) to fully automated vehicles (level-5). Between the levels of no automation to full automation, the automated system has an authority to control the vehicle. In this way, the drivers reduce attention to the road, and, consequently, get distracted as they feel the freedom of disengaging themselves from driving [242,243]. Although the vehicle manufacturing companies and the traffic control agencies clearly state that human drivers should monitor the driving environment at these levels, several challenges related to use and application still persist. Specifically, can a driver remain disengaged from the driving while relying on the ADAS and still maintain a safe driving environment? Similarly, what if the automated system has only the option to save either vehicle or property? Satisfactory answers to these questions are still unclear and belong to an area of active research. 

The authors believe that the mass adoption of eye and gaze trackers depends on their cost as much as their accurate functionality in natural environments (i.e., changing light conditions and usual head movements). In this regard, requirements and features of future eye and gaze trackers are discussed here. 

**Cost:** The prices of existing eye trackers are too high to be used by the general public. The high cost of eye trackers is mainly due to the cost of parts (e.g., high quality lenses and cameras), the development cost, and comparatively limited market. To overcome this problem, the future eye and gaze trackers should opt for the commonly available standard off-the-shelf components, such as digital or web cameras. Additionally, new theoretical and experimental developments are needed so that accurate eye and gaze tracking may be achieved with low quality images.

**Flexibility:** Existing gaze trackers typically need calibration of both the geometric arrangement and the camera(s) which is a tedious job. In certain situations, it could be appropriate to calibrate, for example, the monitor and light sources without requiring the geometric and camera calibration. Such a flexible setup is advantageous for the eye trackers intended for on-the-move usage.

**Calibration:** The present gaze tracking techniques either use a simple prior model with several calibration points or a strong prior model (hardware calibrated) with a brief calibration session. A future direction in gaze tracking is to develop the techniques that require no (or extremely minimal) calibration. We believe that novel eye and gaze models should be developed to realize calibration-free gaze tracking, which is reliable as well.

**Tolerance:** Currently, only partial solutions exist to accommodate the tolerance required by the application involving eyeglasses and contact lenses. The problems in such situations may be partially solved by using multiple light sources coordinated with the users’ head movement relative to the light source and camera. The trend of producing low-cost eye tracking systems may increase for their use in mainstream applications. This practice, however, can lead to low accuracy gaze tracking which could be acceptable for certain applications, but not for ADAS. We believe that additional modeling approaches such as modeling eyeglasses themselves under various light conditions may be required if eye trackers are to be utilized in outdoor applications.

**Interpretation of gaze:** While addressing the technical issues associated with eyes and gaze tracking, the interpretation of relationship between visual and cognitive states is also very important. The analysis of the behavior of eye movements helps determining the cognitive and emotional states as well as the human visual perception. The future eye and gaze trackers may exploit a combination of eye and gaze data with other gestures. Definitely, this is a topic of long-term multi-disciplinary research. 

**Usage of IR and Outdoor Application:** IR light is used in eye tracking systems as it is invisible to the user and light conditions can be controlled to obtain stable gaze estimation and high contrast images. A practical drawback of such systems is the limited reliability when used in outdoor applications. So, the increased reliability in outdoor usage is a requirement for future eye tracking systems. The current efforts to overcome this limitation are in development stages and further research is required. 

**Head mounts:** A part of the research community emphasizes remote gaze tracking, eliminating the need for head mounts. However, the gaze trackers with head mounts may see a revival due to the problems associated with remote trackers and due to the higher attention on portable, tiny head-mounted displays [244]. The head-mounted eye tracking systems are usually more precise as they remain minimally affected by the external variations and their geometry allows for more constraints to be applied. 

## Figures and Tables

**Table 1:** Summary and comparison of eye detection techniques.

Technique	Information					Illumination			Robustness			Requirements				References
	Pupil	Iris	Corner	Eye	Between-the-Eyes	Indoor	Outdoor	Infrared	Scale	Head Pose	Occlusion	High Resolution	High Contrast	Temporal Dependent	Good Initialization	
Shape-based (circular)	✓							✓	✓	✓		✓	✓			[30,31,34]
Shape-based (elliptical)	✓	✓				✓	✓		✓	✓			✓			[27,28,72]
Shape-based (elliptical)	✓							✓	✓	✓			✓	✓		[73,74,75]
Shape-based (complex)	✓	✓	✓			✓	✓		✓	✓		✓			✓	[35,39,41,76]
Feature-based		✓	✓			✓										[42,77]
Feature-based		✓				✓							✓			[62,78]
Feature-based		✓	✓			✓							✓			[50,51]
Feature-based				✓		✓							✓			[53,68,70]
Feature-based					✓	✓			✓	✓	✓					[46,47,48]
Feature-based	✓					✓							✓			[52,60,79,80,81]
Appearance-based				✓		✓	✓		✓	✓						[82,83,84,85]
Symmetry	✓	✓							✓							[57,58,86]
Eye motion				✓		✓	✓							✓		[60,61,62]
Hybrid	✓	✓	✓	✓		✓	✓	✓	✓	✓					✓	[65,69,85]

**Table 2:** Comparison of gaze estimation methods.

No. of Cameras	No. of Lights	Gaze Information	Head Pose Invariant?	Calibration	Accuracy (Degrees)	Comments	References
1	0	PoR	No. Needs extra unit.		2–4	webcam	[27,69,114]
1	0	LoS/LoG	No. Needs extra unit.	Fully	1–2		[90,97,108]
1	0	LoG	Approximate solution		< 1	additional markers, iris radius, parallel with screen	[30]
1	1	PoR	No. Needs extra unit.		1–2	Polynomial approximation	[73,119,120]
1	2	PoR	Yes	Fully	1–3		[88,104,105]
1 + 1 PT camera	1	PoR	Yes	Fully	3	Mirrors	[107]
1 + 1 PT camera	4	PoR	Yes		<2.5	PT camera used during implementation	[95,98]
2	0	PoR	Yes		1	3D face model	[106]
2 + 1 PT camera	1	LoG	Yes		0.7–1		[134]
2 + 2 PT cameras	2	PoR	Yes	Fully	0.6		[99]
2	2(3)	PoR	Yes	Fully	<2	extra lights used during implementation, experimentation conducted with three glints	[96,102]
3	2	PoR	Yes	Fully	not reported		[100,135]
1	1	PoR	No. Needs extra unit.		0.5–1.5	Appearance-based	[68,89,128]

**Table 3:** Summary of measurement techniques.

Measurement	Ability to Detect Distraction			Pros	Cons
	Visual	Cognitive	Visual and Cognitive		
Driving Performance	Y	N	N	Ability to indicate the effect of driving distraction	Requirement of complementary subjective reports to obtain high accuracy results
Physical Measurements	Y	Y	N	Ability to distinguish between distraction types	Unable to distinguish a combined type of distraction
Biological Measurements	Y	Y	Y	Ability to measure cognitive and visual distraction	Intrusiveness
Subjective Reports	N	Y	N	Ability to distinguish underlying mechanism of distraction	Requires input of an expert
Hybrid Measurements	Y	Y	Y	Higher accuracy for discriminating types of distractions Able to complement the blind spots of other methods	Synchronization of multiple source of data with different sampling rate

**Table 4:** Summary of various eye tracking algorithms.

Eye Detection	Tracking Method	Used Features	Algorithm for Distraction/Fatigue Detection	Performance	References
Imaging in the IR spectrum and verification by SVM	Combination of Kalman filter and mean shift	PERCLOS, Head nodding, Head orientation, Eye blink speed, Gaze direction Eye saccadic movement, Yawning	Probability theory (Bayesian network)	Very good	[204]
Imaging in the IR Spectrum	Adaptive filters (Kalman filter)	PERCLOS, Eye blink speed, Gaze direction, Head rotation	Probability theory (Bayesian network)	Very Good	[113]
Imaging in the IR Spectrum	Adaptive filters (Kalman filter)	PERCLOS, Eye blink rate, Eye saccadic movement, Head nodding, Head orientation	Knowledge-based (Fuzzy expert system)		[226]
Feature-based (binarization)	Combination of 4 hierarchical tracking method	PERCLOS, Eye blink rate, Gaze direction, Yawning, Head orientation	Knowledge-based (Finite State Machine)	Average	[227]
Explicitly by Feature-based (projection)	Search window (based on face template matching)	PERCLOS Distance between eyelids, Eye blink rate, Head orientation	Knowledge-based (Fuzzy expert System)	Good	[228]
Other methods (elliptical model in daylight and IR imaging in nightlight)	Combination of NN and condensation algorithm	PERCLOS, Eye blink rate, Head orientation	Thresholding	Good	[229]
Feature-based (projection)	Search window (based on face template matching)	PERCLOS, Distance between eyelids	Thresholding	Good	[230]
Feature-based (projection)	Adaptive filters (UKF)	Continuous eye closure	Thresholding	Average	[231]
Feature-based (projection and connected component analysis)	Search window (eye template matching)	Eyelid distance	Thresholding	Very good	[232]
Feature-based (projection)	Adaptive filters (Kalman filter)	Eye blink rate		Poor	[233]
Feature-based (variance projection and face model)	Adaptive filters (Kalman filter)	PERCLOS, Eye blink speed, Head rotation		Poor	[234]

**Table 5:** A summary of features offered in modern vehicles.

Make	Technology Brand	Description	Alarm Type	Reference
Audi	Rest recommendation system + Audi pre sense	Uses features extracted with the help of far infrared system, camera, radar, thermal camera, lane position, proximity detection to offer features such as collision avoidance assist sunroof and windows closinghigh beam assistturn assistrear cross-path assistexit assist (to warn door opening when a nearby car passes) traffic jam assistnight vision	Audio, display, vibration	[235]
BMW	Active Driving Assistant with Attention Assistant	Uses features extracted with the help of radar, camera, thermal camera, lane position, proximity detection to offer features such as lane change warning, night vision, steering and lane control system for semi-automated driving, crossroad warning, assistive parking	Audio, display, vibration	[236]
Cadillac	Cadillac Super Cruise	System based on FOVIO vision technology developed by Seeing Machines IR camera on the steering wheel column to accurately determine the driver’s attention state	Audio and visual	[237]
Ford	Ford Safe and Smart (Driver alert control)	Uses features extracted with the help of radar, camera, steering sensors, lane position, proximity detection to offer features such as lane-keeping system, adaptive cruise control, forward collision warning with brake support, front rain-sensing windshield wipers, auto high-beam headlamps, blind spot information system, reverse steering	Audio, display, vibration	[238]
Mercedez-Benz	MB Pre-safe Technology	Uses features extracted with the help of radar, camera, sensors on the steering column, steering wheel movement and speed to offer features such as driver’s profile and behaviour, accident investigation, pre-safe brake and distronic plus technology, night view assist plus, active lane keeping assist and active blind spot monitoring, adaptive high beam assist, attention assist	Audio, display	[239]
Toyota	Toyota Safety Sense	Uses features extracted with the help of radar, charge-coupled camera, eye tracking and head motion, audio, display advanced obstacle detection system, pre-collision system, lane departure alert, automatic high beams, dynamic radar cruise control, pedestrian detection,	Audio, display	[240]

## References

[1] Omer Y., Sapir R., Hatuka Y., Yovel G. (2019). What Is a Face? Critical Features for Face Detection. Perception.

[2] Cho S.W., Baek N.R., Kim M.C., Koo J.H., Kim J.H., Park K.R. (2018). Face Detection in Nighttime Images Using Visible-Light Camera Sensors with Two-Step Faster Region-Based Convolutional Neural Network. Sensors.

[3] Bozomitu R.G., Păsărică A., Tărniceriu D., Rotariu C. (2019). Development of an Eye Tracking-Based Human-Computer Interface for Real-Time Applications. Sensors.

[4] Cornia M., Baraldi L., Serra G., Cucchiara R. (2018). Predicting Human Eye Fixations via an LSTM-Based Saliency Attentive Model. IEEE Trans. Image Process..

[5] Huey E.B. (1908). The Psychology and Pedagogy of Reading.

[6] Buswell G.T. (1922). Fundamental Reading Habits: A Study of Their Development.

[7] Buswell G.T. (1935). How People Look at Pictures: A Study of the Psychology and Perception in Art.

[8] Yarbus A.L. (2013). Eye Movements and Vision.

[9] Rayner K. (1978). Eye movements in reading and information processing. Psychol. Bull..

[10] Wright R.D., Ward L.M. (2008). Orienting of Attention.

[11] Posner M.I. (1980). Orienting of attention. Q. J. Exp. Psychol..

[12] Carpenter P.A., Just M.A. (1983). What your eyes do while your mind is reading. Eye Movements in Reading.

[13] Jacob R.J., Karn K.S. (2003). Eye tracking in human-computer interaction and usability research: Ready to deliver the promises. The Mind’s Eye.

[14] Aleem I.S., Vidal M., Chapeskie J. (2018). Systems, Devices, and Methods for Laser Eye Tracking. U.S. Patent.

[15] Naqvi R.A., Arsalan M., Batchuluun G., Yoon H.S., Park K.R. (2018). Deep Learning-Based Gaze Detection System for Automobile Drivers Using a NIR Camera Sensor. Sensors.

[16] Swaminathan A., Ramachandran M. (2018). Enabling Augmented Reality Using Eye Gaze Tracking. U.S. Patent.

[17] Vicente F., Huang Z., Xiong X., De la Torre F., Zhang W., Levi D. (2015). Driver gaze tracking and eyes off the road detection system. IEEE Trans. Intell. Transp. Syst..

[18] Massé B., Ba S., Horaud R. (2017). Tracking gaze and visual focus of attention of people involved in social interaction. IEEE Trans. Pattern Anal. Mach. Intell..

[19] Ramirez Gomez A., Lankes M. (2019). Towards Designing Diegetic Gaze in Games: The Use of Gaze Roles and Metaphors. Multimodal Technol. Interact..

[20] World Health Organization (2015). Global Status Report on Road Safety 2013.

[21] World Health Organization (2014). World Report on Road Traffic Injury Prevention.

[22] World Health Organization (2009). Global Status Report on Road Safety: Time for Action.

[23] Bayly M., Fildes B., Regan M., Young K. (2007). Review of crash effectiveness of intelligent transport systems. Emergency.

[24] Society of Automotive Engineers (2015). Operational Definitions of Driving Performance Measures and Statistics.

[25] Kiefer P., Giannopoulos I., Raubal M., Duchowski A. (2017). Eye tracking for spatial research: Cognition, computation, challenges. Spat. Cogn. Comput..

[26] Topolšek D., Areh I., Cvahte T. (2016). Examination of driver detection of roadside traffic signs and advertisements using eye tracking. Transp. Res. F Traffic Psychol. Behav..

[27] Hansen D.W., Pece A.E.C. (2005). Eye tracking in the wild. Comput. Vis. Image Underst..

[28] Daugman J. (2003). The importance of being random: Statistical principles of iris recognition. Pattern Recognit..

[29] Young D., Tunley H., Samuels R. (1995). Specialised Hough Transform and Active Contour Methods for Real-Time Eye Tracking.

[30] Kyung-Nam K., Ramakrishna R.S. Vision-based eye-gaze tracking for human computer interface. Proceedings of the IEEE SMC’99 Conference Proceedings, IEEE International Conference on Systems, Man, and Cybernetics.

[31] Peréz A., Córdoba M.L., Garcia A., Méndez R., Munoz M., Pedraza J.L., Sanchez F. (2003). A Precise Eye-Gaze Detection and Tracking System.

[32] Comaniciu D., Ramesh V., Meer P. (2003). Kernel-based object tracking. IEEE Trans. Pattern Anal. Mach. Intell..

[33] Locher P.J., Nodine C.F., O’Regan J.K., Levy-Schoen A. (1987). Symmetry Catches the Eye. Eye Movements from Physiology to Cognition.

[34] Dongheng L., Winfield D., Parkhurst D.J. Starburst: A hybrid algorithm for video-based eye tracking combining feature-based and model-based approaches. Proceedings of the IEEE Computer Society Conference on Computer Vision and Pattern Recognition (CVPR’05)-Workshops.

[35] Yuille A.L., Hallinan P.W., Cohen D.S. (1992). Feature extraction from faces using deformable templates. Int. J. Comput. Vision.

[36] Kin-Man L., Hong Y. (1996). Locating and extracting the eye in human face images. Pattern Recognit..

[37] Edwards G.J., Cootes T.F., Taylor C.J. (1998). Face recognition using active appearance models. European Conference on Computer Vision—ECCV’98.

[38] Heo H., Lee W.O., Shin K.Y., Park K.R. (2014). Quantitative Measurement of Eyestrain on 3D Stereoscopic Display Considering the Eye Foveation Model and Edge Information. Sensors.

[39] Zhang L. Estimation of eye and mouth corner point positions in a knowledge-based coding system. Proceedings of the Digital Compression Technologies and Systems for Video Communications.

[40] Kampmann M., Zhang L. Estimation of eye, eyebrow and nose features in videophone sequences. Proceedings of the International Workshop on Very Low Bitrate Video Coding (VLBV 98).

[41] Chow G., Li X. (1993). Towards a system for automatic facial feature detection. Pattern Recognit..

[42] Herpers R., Michaelis M., Lichtenauer K., Sommer G. Edge and keypoint detection in facial regions. Proceedings of the Second International Conference on Automatic Face and Gesture Recognition.

[43] Li B., Fu H., Wen D., Lo W. (2018). Etracker: A Mobile Gaze-Tracking System with Near-Eye Display Based on a Combined Gaze-Tracking Algorithm. Sensors.

[44] Vincent J.M., Waite J.B., Myers D.J. (1992). Automatic location of visual features by a system of multilayered perceptrons. IEE Proc. F Radar Signal Process..

[45] Reinders M.J.T., Koch R.W.C., Gerbrands J.J. Locating facial features in image sequences using neural networks. Proceedings of the Second International Conference on Automatic Face and Gesture Recognition.

[46] Kawato S., Ohya J. Two-step approach for real-time eye tracking with a new filtering technique. Proceedings of the Smc Conference Proceedings, IEEE International Conference on Systems, Man and Cybernetics. ‘Cybernetics Evolving to Systems, Humans, Organizations, and Their Complex Interactions’ (Cat. No. 0).

[47] Kawato S., Ohya J. Real-time detection of nodding and head-shaking by directly detecting and tracking the “between-eyes”. Proceedings of the Fourth IEEE International Conference on Automatic Face and Gesture Recognition.

[48] Kawato S., Tetsutani N. (2004). Detection and tracking of eyes for gaze-camera control. Image Vis. Comput..

[49] Kawato S., Tetsutani N. Real-time detection of between-the-eyes with a circle frequency filter. Proceedings of the 5th Asian Conference on Computer Vision (ACCV2002).

[50] Sirohey S., Rosenfeld A., Duric Z. (2002). A method of detecting and tracking irises and eyelids in video. Pattern Recognit..

[51] Sirohey S.A., Rosenfeld A. (2001). Eye detection in a face image using linear and nonlinear filters. Pattern Recognit..

[52] Yang J., Stiefelhagen R., Meier U., Waibel A. Real-time face and facial feature tracking and applications. Proceedings of the AVSP’98 International Conference on Auditory-Visual Speech Processing.

[53] Ying-li T., Kanade T., Cohn J.F. Dual-state parametric eye tracking. Proceedings of the Fourth IEEE International Conference on Automatic Face and Gesture Recognition.

[54] Lucas B.D., Kanade T. An iterative image registration technique with an application to stereo vision. Proceedings of the 7th International Joint Conference on Artificial Intelligence-Volume 2.

[55] Weimin H., Mariani R. Face detection and precise eyes location. Proceedings of the 15th International Conference on Pattern Recognition, ICPR-2000.

[56] Samaria F., Young S. (1994). HMM-based architecture for face identification. Image Vis. Comput..

[57] Kovesi P. Symmetry and asymmetry from local phase. Proceedings of the Tenth Australian Joint Conference on Artificial Intelligence.

[58] Lin C.-C., Lin W.-C. (1996). Extracting facial features by an inhibitory mechanism based on gradient distributions. Pattern Recognit..

[59] Sela G., Levine M.D. (1997). Real-Time Attention for Robotic Vision. Real Time Imaging.

[60] Grauman K., Betke M., Gips J., Bradski G.R. Communication via eye blinks-detection and duration analysis in real time. Proceedings of the 2001 IEEE Computer Society Conference on Computer Vision and Pattern Recognition, CVPR 2001.

[61] Crowley J.L., Berard F. Multi-modal tracking of faces for video communications. Proceedings of the IEEE Computer Society Conference on Computer Vision and Pattern Recognition.

[62] Bala L.-P. Automatic detection and tracking of faces and facial features in video sequences. Proceedings of the Picture Coding Symposium.

[63] Ishikawa T. (2004). Passive Driver Gaze Tracking with Active Appearance Models.

[64] Matsumoto Y., Zelinsky A. An algorithm for real-time stereo vision implementation of head pose and gaze direction measurement. Proceedings of the Fourth IEEE International Conference on Automatic Face and Gesture Recognition (Cat. No. PR00580).

[65] Xie X., Sudhakar R., Zhuang H. (1994). On improving eye feature extraction using deformable templates. Pattern Recognit..

[66] Feris R.S., de Campos T.E., Marcondes R.C. (2000). Detection and Tracking of Facial Features in Video Sequences.

[67] Horng W.-B., Chen C.-Y., Chang Y., Fan C.-H. Driver fatigue detection based on eye tracking and dynamic template matching. Proceedings of the IEEE International Conference on Networking, Sensing and Control.

[68] Stiefelhagen R., Yang J., Waibel A. Tracking eyes and monitoring eye gaze. Proceedings of the Workshop on Perceptual User Interfaces.

[69] Hansen D.W., Hansen J.P., Nielsen M., Johansen A.S., Stegmann M.B. Eye typing using Markov and active appearance models. Proceedings of the Sixth IEEE Workshop on Applications of Computer Vision (WACV 2002).

[70] Stiefelhagen R., Jie Y., Waibel A. A model-based gaze tracking system. Proceedings of the IEEE International Joint Symposia on Intelligence and Systems.

[71] Xie X., Sudhakar R., Zhuang H. (1998). A cascaded scheme for eye tracking and head movement compensation. IEEE Trans. Syst. Man Cybern. Part A Syst. Hum..

[72] Valenti R., Gevers T. Accurate eye center location and tracking using isophote curvature. Proceedings of the 2008 IEEE Conference on Computer Vision and Pattern Recognition.

[73] Morimoto C.H., Koons D., Amir A., Flickner M. (2000). Pupil detection and tracking using multiple light sources. Image Vis. Comput..

[74] Morimoto C.H., Flickner M. Real-time multiple face detection using active illumination. Proceedings of the Fourth IEEE International Conference on Automatic Face and Gesture Recognition (Cat. No. PR00580).

[75] Ebisawa Y. Realtime 3D position detection of human pupil. Proceedings of the 2004 IEEE Symposium on Virtual Environments, Human-Computer Interfaces and Measurement Systems (VCIMS).

[76] Colombo C., Del Bimbo A. (1999). Real-time head tracking from the deformation of eye contours using a piecewise affine camera. Pattern Recognit. Lett..

[77] Feng G.C., Yuen P.C. (1998). Variance projection function and its application to eye detection for human face recognition. Pattern Recognit. Lett..

[78] Orazio T.D., Leo M., Cicirelli G., Distante A. An algorithm for real time eye detection in face images. Proceedings of the 17th International Conference on Pattern Recognition, ICPR 2004.

[79] Hallinan P.W. (1991). Recognizing Human Eyes.

[80] Hillman P.M., Hannah J.M., Grant P.M. Global fitting of a facial model to facial features for model-based video coding. Proceedings of the 3rd International Symposium on Image and Signal Processing and Analysis, ISPA 2003.

[81] Zhu Z., Fujimura K., Ji Q. Real-time eye detection and tracking under various light conditions. Proceedings of the 2002 Symposium on Eye Tracking Research & Applications.

[82] Fasel I., Fortenberry B., Movellan J. (2005). A generative framework for real time object detection and classification. Comput. Vis. Image Underst..

[83] Huang J., Wechsler H. Eye location using genetic algorithm. Proceedings of the 2nd International Conference on Audio and Video-Based Biometric Person Authentication.

[84] Hansen D.W., Hammoud R.I. (2007). An improved likelihood model for eye tracking. Comput. Vis. Image Underst..

[85] Cristinacce D., Cootes T.F. Feature detection and tracking with constrained local models. Proceedings of the British Machine Vision Conference.

[86] Kimme C., Ballard D., Sklansky J. (1975). Finding circles by an array of accumulators. Commun. ACM.

[87] Ruddock K.H. (1989). Movements of the Eyes. J. Mod. Opt..

[88] Guestrin E.D., Eizenman M. (2006). General theory of remote gaze estimation using the pupil center and corneal reflections. IEEE Trans. Biomed. Eng..

[89] Baluja S., Pomerleau D. (1994). Non-Intrusive Gaze Tracking Using Artificial Neural Networks.

[90] Wang J.-G., Sung E., Venkateswarlu R. (2005). Estimating the eye gaze from one eye. Comput. Vis. Image Underst..

[91] Fridman L., Lee J., Reimer B., Victor T. (2016). ‘Owl’ and ‘Lizard’: Patterns of head pose and eye pose in driver gaze classification. IET Computer Vision.

[92] Ohno T. One-point calibration gaze tracking method. Proceedings of the 2006 Symposium on Eye Tracking Research & Applications.

[93] Ohno T., Mukawa N., Yoshikawa A. FreeGaze: A gaze tracking system for everyday gaze interaction. Proceedings of the 2002 Symposium on Eye Tracking Research & Applications.

[94] Villanueva A., Cabeza R. (2007). Models for Gaze Tracking Systems. EURASIP J. Image Video Process..

[95] Coutinho F.L., Morimoto C.H. Free head motion eye gaze tracking using a single camera and multiple light sources. Proceedings of the 2006 19th Brazilian Symposium on Computer Graphics and Image Processing.

[96] Shih S.W., Wu Y.T., Liu J. A calibration-free gaze tracking technique. Proceedings of the 15th International Conference on Pattern Recognition, ICPR-2000.

[97] Villanueva A., Cabeza R., Porta S. (2006). Eye tracking: Pupil orientation geometrical modeling. Image Vis. Comput..

[98] Yoo D.H., Chung M.J. (2005). A novel non-intrusive eye gaze estimation using cross-ratio under large head motion. Comput. Vis. Image Underst..

[99] Beymer D., Flickner M. Eye gaze tracking using an active stereo head. Proceedings of the 2003 IEEE Computer Society Conference on Computer Vision and Pattern Recognition.

[100] Brolly X.L.C., Mulligan J.B. Implicit Calibration of a Remote Gaze Tracker. Proceedings of the 2004 Conference on Computer Vision and Pattern Recognition Workshop.

[101] Ohno T., Mukawa N. A free-head, simple calibration, gaze tracking system that enables gaze-based interaction. Proceedings of the 2004 symposium on Eye Tracking Research & Applications.

[102] Shih S.-W., Liu J. (2004). A novel approach to 3-D gaze tracking using stereo cameras. Trans. Sys. Man Cyber. Part B.

[103] Kim S.M., Sked M., Ji Q. Non-intrusive eye gaze tracking under natural head movements. Proceedings of the 26th Annual International Conference of the IEEE Engineering in Medicine and Biology Society.

[104] Meyer A., Böhme M., Martinetz T., Barth E. (2006). A Single-Camera Remote Eye Tracker. International Tutorial and Research Workshop on Perception and Interactive Technologies for Speech-Based Systems.

[105] Morimoto C.H., Amir A., Flickner M. Detecting eye position and gaze from a single camera and 2 light sources. Proceedings of the Object Recognition Supported by User Interaction for Service Robots.

[106] Newman R., Matsumoto Y., Rougeaux S., Zelinsky A. Real-time stereo tracking for head pose and gaze estimation. Proceedings of the Fourth IEEE International Conference on Automatic Face and Gesture Recognition (Cat. No. PR00580).

[107] Noureddin B., Lawrence P.D., Man C.F. (2005). A non-contact device for tracking gaze in a human computer interface. Comput. Vis. Image Underst..

[108] Villanueva A., Cabeza R., Porta S. (2007). Gaze Tracking system model based on physical parameters. Int. J. Pattern Recognit. Artif. Intell..

[109] Hansen D.W., Skovsgaard H.H.T., Hansen J.P., Møllenbach E. Noise tolerant selection by gaze-controlled pan and zoom in 3D. Proceedings of the 2008 Symposium on Eye Tracking Research & Applications.

[110] Vertegaal R., Weevers I., Sohn C. GAZE-2: An attentive video conferencing system. Proceedings of the CHI’02 Extended Abstracts on Human Factors in Computing Systems.

[111] Ebisawa Y., Satoh S. Effectiveness of pupil area detection technique using two light sources and image difference method. Proceedings of the 15th Annual International Conference of the IEEE Engineering in Medicine and Biology Societ.

[112] Morimoto C.H., Mimica M.R.M. (2005). Eye gaze tracking techniques for interactive applications. Comput. Vis. Image Underst..

[113] Ji Q., Yang X. (2002). Real-Time Eye, Gaze, and Face Pose Tracking for Monitoring Driver Vigilance. Real Time Imaging.

[114] Williams O., Blake A., Cipolla R. Sparse and Semi-supervised Visual Mapping with the S^ 3GP. Proceedings of the 2006 IEEE Computer Society Conference on Computer Vision and Pattern Recognition (CVPR’06).

[115] Bin Suhaimi M.S.A., Matsushita K., Sasaki M., Njeri W. (2019). 24-Gaze-Point Calibration Method for Improving the Precision of AC-EOG Gaze Estimation. Sensors.

[116] Hansen D.W. (2003). Committing Eye Tracking.

[117] Stampe D.M. (1993). Heuristic filtering and reliable calibration methods for video-based pupil-tracking systems. Behav. Res. Methods Instrum. Comput..

[118] Merchant J., Morrissette R., Porterfield J.L. (1974). Remote Measurement of Eye Direction Allowing Subject Motion Over One Cubic Foot of Space. IEEE Trans. Biomed. Eng..

[119] White K.P., Hutchinson T.E., Carley J.M. (1993). Spatially dynamic calibration of an eye-tracking system. IEEE Trans. Syst. Man Cybern..

[120] Zhu Z., Ji Q. (2004). Eye and gaze tracking for interactive graphic display. Mach. Vis. Appl..

[121] Zhiwei Z., Qiang J., Bennett K.P. Nonlinear Eye Gaze Mapping Function Estimation via Support Vector Regression. Proceedings of the 18th International Conference on Pattern Recognition (ICPR’06).

[122] Kolakowski S.M., Pelz J.B. Compensating for eye tracker camera movement. Proceedings of the 2006 Symposium on Eye Tracking Research & Applications.

[123] Zhu Z., Ji Q. (2007). Novel Eye Gaze Tracking Techniques Under Natural Head Movement. IEEE Trans. Biomed. Eng..

[124] Müller P.U., Cavegn D., d’Ydewalle G., Groner R. (1993). A comparison of a new limbus tracker, corneal reflection technique, Purkinje eye tracking and electro-oculography. Perception and Cognition: Advances in Eye Movement Research.

[125] Crane H.D., Steele C.M. (1978). Accurate three-dimensional eyetracker. Appl. Opt..

[126] Xu L.-Q., Machin D., Sheppard P. A Novel Approach to Real-time Non-intrusive Gaze Finding. Proceedings of the British Machine Vision Conference.

[127] Wang J.-G., Sung E. (2001). Gaze determination via images of irises. Image Vis. Comput..

[128] Tan K.-H., Kriegman D.J., Ahuja N. Appearance-based eye gaze estimation. Proceedings of the Sixth IEEE Workshop on Applications of Computer Vision (WACV 2002).

[129] Heinzmann K., Zelinsky A. 3-D Facial Pose and Gaze Point Estimation Using a Robust Real-Time Tracking Paradigm. Proceedings of the 3rd, International Conference on Face & Gesture Recognition.

[130] Yamazoe H., Utsumi A., Yonezawa T., Abe S. Remote gaze estimation with a single camera based on facial-feature tracking without special calibration actions. Proceedings of the 2008 Symposium on Eye Tracking Research & Applications.

[131] Matsumoto Y., Ogasawara T., Zelinsky A. Behavior recognition based on head pose and gaze direction measurement. Proceedings of the 2000 IEEE/RSJ International Conference on Intelligent Robots and Systems (IROS 2000) (Cat. No.00CH37113).

[132] Li G., Li S.E., Zou R., Liao Y., Cheng B. (2019). Detection of road traffic participants using cost-effective arrayed ultrasonic sensors in low-speed traffic situations. Mech. Syst. Signal Process..

[133] Scott D., Findlay J.M., Hursley Human Factors Laboratory W., Laboratory I.U.H.H.F. (1991). Visual Search, Eye Movements and Display Units.

[134] Talmi K., Liu J. (1999). Eye and gaze tracking for visually controlled interactive stereoscopic displays. Signal Process. Image Commun..

[135] Tomono A., Iida M., Kobayashi Y. (1990). A TV Camera System Which Extracts Feature Points for Non-Contact Eye Movement Detection.

[136] Harbluk J.L., Noy Y.I., Eizenman M. (2002). The Impact of Cognitive Distraction on Driver Visual Behaviour and Vehicle Control.

[137] Sodhi M., Reimer B., Cohen J.L., Vastenburg E., Kaars R., Kirschenbaum S. On-road driver eye movement tracking using head-mounted devices. Proceedings of the 2002 Symposium on Eye Tracking Research & Applications.

[138] Reimer B., Mehler B., Wang Y., Coughlin J.F. (2012). A Field Study on the Impact of Variations in Short-Term Memory Demands on Drivers’ Visual Attention and Driving Performance Across Three Age Groups. Hum. Factors.

[139] Reimer B., Mehler B., Wang Y., Coughlin J.F. (2010). The Impact of Systematic Variation of Cognitive Demand on Drivers’ Visual Attention across Multiple Age Groups. Proc. Hum. Factors Ergon. Soc. Annu. Meet..

[140] Liang Y., Lee J.D. (2010). Combining cognitive and visual distraction: Less than the sum of its parts. Accid. Anal. Prev..

[141] Harbluk J.L., Noy Y.I., Trbovich P.L., Eizenman M. (2007). An on-road assessment of cognitive distraction: Impacts on drivers’ visual behavior and braking performance. Accid. Anal. Prev..

[142] Victor T.W., Harbluk J.L., Engström J.A. (2005). Sensitivity of eye-movement measures to in-vehicle task difficulty. Transp. Res. Part F Traffic Psychol. Behav..

[143] Recarte M.A., Nunes L.M. (2000). Effects of verbal and spatial-imagery tasks on eye fixations while driving. J. Exp. Psychol. Appl..

[144] Recarte M.A., Nunes L.M. (2003). Mental workload while driving: Effects on visual search, discrimination, and decision making. J. Exp. Psychol. Appl..

[145] Nunes L., Recarte M.A. (2002). Cognitive demands of hands-free-phone conversation while driving. Transp. Res. Part F Traffic Psychol. Behav..

[146] Kass S.J., Cole K.S., Stanny C.J. (2007). Effects of distraction and experience on situation awareness and simulated driving. Transp. Res. Part F Traffic Psychol. Behav..

[147] Engström J., Johansson E., Östlund J. (2005). Effects of visual and cognitive load in real and simulated motorway driving. Transp. Res. Part F Traffic Psychol. Behav..

[148] Ahlström C., Kircher K., Kircher A. Considerations when calculating percent road centre from eye movement data in driver distraction monitoring. Proceedings of the Fifth International Driving Symposium on Human Factors in Driver Assessment, Training and Vehicle Design.

[149] Wang Y., Reimer B., Dobres J., Mehler B. (2014). The sensitivity of different methodologies for characterizing drivers’ gaze concentration under increased cognitive demand. Transp. Res. F Traffic Psychol. Behav..

[150] Endsley M.R. (2016). Toward a Theory of Situation Awareness in Dynamic Systems.

[151] Khan M.Q., Lee S. (2019). A Comprehensive Survey of Driving Monitoring and Assistance Systems. Sensors.

[152] Martinez C.M., Heucke M., Wang F., Gao B., Cao D. (2018). Driving Style Recognition for Intelligent Vehicle Control and Advanced Driver Assistance: A Survey. IEEE Trans. Intell. Transp. Syst..

[153] Regan M.A., Lee J.D., Young K. (2008). Driver Distraction: Theory, Effects, and Mitigation.

[154] Ranney T., Mazzae E., Garrott R., Goodman M., Administration N.H.T.S. Driver distraction research: Past, present and future. Proceedings of the 17th International Technical Conference of Enhanced Safety of Vehicles.

[155] Young K., Regan M., Hammer M. (2007). Driver distraction: A review of the literature. Distracted Driv..

[156] Stutts J.C., Reinfurt D.W., Staplin L., Rodgman E. (2001). The Role of Driver Distraction in Traffic Crashes.

[157] Zhao Y., Görne L., Yuen I.-M., Cao D., Sullman M., Auger D., Lv C., Wang H., Matthias R., Skrypchuk L. (2017). An Orientation Sensor-Based Head Tracking System for Driver Behaviour Monitoring. Sensors.

[158] Khandakar A., Chowdhury M.E.H., Ahmed R., Dhib A., Mohammed M., Al-Emadi N.A.M.A., Michelson D. (2019). Portable System for Monitoring and Controlling Driver Behavior and the Use of a Mobile Phone While Driving. Sensors.

[159] Ranney T.A., Garrott W.R., Goodman M.J. (2001). NHTSA Driver Distraction Research: Past, Present, and Future.

[160] Fitch G.M., Soccolich S.A., Guo F., McClafferty J., Fang Y., Olson R.L., Perez M.A., Hanowski R.J., Hankey J.M., Dingus T.A. (2013). The Impact of Hand-Held and Hands-Free Cell Phone Use on Driving Performance and Safety-Critical Event Risk.

[161] Miller J., Ulrich R. (2008). Bimanual Response Grouping in Dual-Task Paradigms. Q. J. Exp. Psychol..

[162] Gazes Y., Rakitin B.C., Steffener J., Habeck C., Butterfield B., Ghez C., Stern Y. (2010). Performance degradation and altered cerebral activation during dual performance: Evidence for a bottom-up attentional system. Behav. Brain Res..

[163] Törnros J.E.B., Bolling A.K. (2005). Mobile phone use—Effects of handheld and handsfree phones on driving performance. Accid. Anal. Prev..

[164] Young K.L., Salmon P.M., Cornelissen M. (2013). Distraction-induced driving error: An on-road examination of the errors made by distracted and undistracted drivers. Accid. Anal. Prev..

[165] Chan M., Singhal A. (2013). The emotional side of cognitive distraction: Implications for road safety. Accid. Anal. Prev..

[166] Strayer D.L., Cooper J.M., Turrill J., Coleman J., Medeiros-Ward N., Biondi F. (2013). Measuring Cognitive Distraction in the Automobile.

[167] Rakauskas M.E., Gugerty L.J., Ward N.J. (2004). Effects of naturalistic cell phone conversations on driving performance. J. Saf. Res..

[168] Horberry T., Anderson J., Regan M.A., Triggs T.J., Brown J. (2006). Driver distraction: The effects of concurrent in-vehicle tasks, road environment complexity and age on driving performance. Accid. Anal. Prev..

[169] Awais M., Badruddin N., Drieberg M. (2017). A Hybrid Approach to Detect Driver Drowsiness Utilizing Physiological Signals to Improve System Performance and Wearability. Sensors.

[170] Chien J.-C., Chen Y.-S., Lee J.-D. (2017). Improving Night Time Driving Safety Using Vision-Based Classification Techniques. Sensors.

[171] Thum Chia C., Mustafa M.M., Hussain A., Hendi S.F., Majlis B.Y. Development of vehicle driver drowsiness detection system using electrooculogram (EOG). Proceedings of the 2005 1st International Conference on Computers, Communications, & Signal Processing with Special Track on Biomedical Engineering.

[172] Sirevaag E.J., Stern J.A. (2000). Ocular Measures of Fatigue and Cognitive Factors. Engineering Psychophysiology: Issues and Applications.

[173] Schleicher R., Galley N., Briest S., Galley L. (2008). Blinks and saccades as indicators of fatigue in sleepiness warnings: Looking tired?. Ergonomics.

[174] Yue C. (2011). EOG Signals in Drowsiness Research. https://pdfs.semanticscholar.org/8b77/9934f6ceae3073b3312c947f39467a74828f.pdf.

[175] Thorslund B. (2004). Electrooculogram Analysis and Development of a System for Defining Stages of Drowsiness.

[176] Pohl J., Birk W., Westervall L. (2007). A driver-distraction-based lane-keeping assistance system. Proc Inst. Mech. Eng. Part I J. Syst. Control Eng..

[177] Kircher K., Ahlstrom C., Kircher A. Comparison of two eye-gaze based real-time driver distraction detection algorithms in a small-scale field operational test. Proceedings of the Fifth International Driving Symposium on Human Factors in Driver Assessment, Training and Vehicle Design.

[178] Kim W., Jung W.-S., Choi H.K. (2019). Lightweight Driver Monitoring System Based on Multi-Task Mobilenets. Sensors.

[179] Mavely A.G., Judith J.E., Sahal P.A., Kuruvilla S.A. Eye gaze tracking based driver monitoring system. Proceedings of the 2017 IEEE International Conference on Circuits and Systems (ICCS).

[180] Wollmer M., Blaschke C., Schindl T., Schuller B., Farber B., Mayer S., Trefflich B. (2011). Online Driver Distraction Detection Using Long Short-Term Memory. IEEE Trans. Intell. Transp. Syst..

[181] Castro M.J.C.D., Medina J.R.E., Lopez J.P.G., Goma J.C.d., Devaraj M. A Non-Intrusive Method for Detecting Visual Distraction Indicators of Transport Network Vehicle Service Drivers Using Computer Vision. Proceedings of the IEEE 10th International Conference on Humanoid, Nanotechnology, Information Technology, Communication and Control, Environment and Management (HNICEM).

[182] Banaeeyan R., Halin A.A., Bahari M. Nonintrusive eye gaze tracking using a single eye image. Proceedings of the 2015 IEEE International Conference on Signal and Image Processing Applications (ICSIPA).

[183] Anjali K.U., Thampi A.K., Vijayaraman A., Francis M.F., James N.J., Rajan B.K. Real-time nonintrusive monitoring and detection of eye blinking in view of accident prevention due to drowsiness. Proceedings of the 2016 International Conference on Circuit, Power and Computing Technologies (ICCPCT).

[184] Hirayama T., Mase K., Takeda K. (2013). Analysis of Temporal Relationships between Eye Gaze and Peripheral Vehicle Behavior for Detecting Driver Distraction. Int. J. Veh. Technol..

[185] Yang Y., Sun H., Liu T., Huang G.-B., Sourina O. (2015). Driver Workload Detection in On-Road Driving Environment Using Machine Learning. Proceedings of ELM-2014 Volume 2.

[186] Tango F., Botta M. (2013). Real-Time Detection System of Driver Distraction Using Machine Learning. IEEE Trans. Intell. Transp. Syst..

[187] Mbouna R.O., Kong S.G., Chun M. (2013). Visual Analysis of Eye State and Head Pose for Driver Alertness Monitoring. IEEE Trans. Intell. Transp. Syst..

[188] Ahlstrom C., Kircher K., Kircher A. (2013). A Gaze-Based Driver Distraction Warning System and Its Effect on Visual Behavior. IEEE Trans. Intell. Transp. Syst..

[189] Liu T., Yang Y., Huang G., Yeo Y.K., Lin Z. (2016). Driver Distraction Detection Using Semi-Supervised Machine Learning. IEEE Trans. Intell. Transp. Syst..

[190] Yekhshatyan L., Lee J.D. (2013). Changes in the Correlation between Eye and Steering Movements Indicate Driver Distraction. IEEE Trans. Intell. Transp. Syst..

[191] Carsten O., Brookhuis K. (2005). Issues arising from the HASTE experiments. Transp. Res. Part F Traffic Psychol. Behav..

[192] Ebrahim P. (2016). Driver Drowsiness Monitoring Using Eye Movement Features Derived from Electrooculography. Ph.D. Thesis.

[193] Shin D.U.K., Sakai H., Uchiyama Y. (2011). Slow eye movement detection can prevent sleep-related accidents effectively in a simulated driving task. J. Sleep Res..

[194] Liang Y., Reyes M.L., Lee J.D. (2007). Real-Time Detection of Driver Cognitive Distraction Using Support Vector Machines. IEEE Trans. Intell. Transp. Syst..

[195] Liang Y., Lee J.D. (2014). A hybrid Bayesian Network approach to detect driver cognitive distraction. Transp. Res. Part C Emerg. Technol..

[196] Weller G., Schlag B. A robust method to detect driver distraction. http://www.humanist-vce.eu/fileadmin/contributeurs/humanist/Berlin2010/4a_Weller.pdf.

[197] Miyaji M., Kawanaka H., Oguri K. Driver’s cognitive distraction detection using physiological features by the adaboost. Proceedings of the 2009 12th International IEEE Conference on Intelligent Transportation Systems.

[198] Xu J., Min J., Hu J. (2018). Real-time eye tracking for the assessment of driver fatigue. Healthc. Technol. Lett..

[199] Tang J., Fang Z., Hu S., Ying S. Driver fatigue detection algorithm based on eye features. Proceedings of the 2010 Seventh International Conference on Fuzzy Systems and Knowledge Discovery.

[200] Li J., Yang Z., Song Y. A hierarchical fuzzy decision model for driver’s unsafe states monitoring. Proceedings of the 2011 Eighth International Conference on Fuzzy Systems and Knowledge Discovery (FSKD).

[201] Rigane O., Abbes K., Abdelmoula C., Masmoudi M. A Fuzzy Based Method for Driver Drowsiness Detection. Proceedings of the IEEE/ACS 14th International Conference on Computer Systems and Applications (AICCSA).

[202] Lethaus F., Baumann M.R., Köster F., Lemmer K. (2011). Using pattern recognition to predict driver intent. International Conference on Adaptive and Natural Computing Algorithms.

[203] Xiao Z., Hu Z., Geng L., Zhang F., Wu J., Li Y. (2019). Fatigue driving recognition network: Fatigue driving recognition via convolutional neural network and long short-term memory units. IET Trans. Intell. Transp. Syst..

[204] Qiang J., Zhiwei Z., Lan P. (2004). Real-time nonintrusive monitoring and prediction of driver fatigue. IEEE Trans. Veh. Technol..

[205] Wang H., Song W., Liu W., Song N., Wang Y., Pan H. (2018). A Bayesian Scene-Prior-Based Deep Network Model for Face Verification. Sensors.

[206] Jain A., Koppula H.S., Soh S., Raghavan B., Singh A., Saxena A. (2016). Brain4cars: Car that knows before you do via sensory-fusion deep learning architecture. arXiv.

[207] Fridman L., Langhans P., Lee J., Reimer B. (2016). Driver Gaze Region Estimation without Use of Eye Movement. IEEE Intell. Syst..

[208] Li G., Yang Y., Qu X. (2019). Deep Learning Approaches on Pedestrian Detection in Hazy Weather. IEEE Trans. Ind. Electron..

[209] Song C., Yan X., Stephen N., Khan A.A. (2018). Hidden Markov model and driver path preference for floating car trajectory map matching. IET Intell. Transp. Syst..

[210] Muñoz M., Reimer B., Lee J., Mehler B., Fridman L. (2016). Distinguishing patterns in drivers’ visual attention allocation using Hidden Markov Models. Transp. Res. Part F Traffic Psychol. Behav..

[211] Hou H., Jin L., Niu Q., Sun Y., Lu M. (2011). Driver Intention Recognition Method Using Continuous Hidden Markov Model. Int. J. Comput. Intell. Syst..

[212] Fu R., Wang H., Zhao W. (2016). Dynamic driver fatigue detection using hidden Markov model in real driving condition. Expert Syst. Appl..

[213] Morris B., Doshi A., Trivedi M. Lane change intent prediction for driver assistance: On-road design and evaluation. Proceedings of the 2011 IEEE Intelligent Vehicles Symposium (IV).

[214] Tang J., Liu F., Zhang W., Ke R., Zou Y. (2018). Lane-changes prediction based on adaptive fuzzy neural network. Expert Syst. Appl..

[215] Zhu W., Miao J., Hu J., Qing L. (2014). Vehicle detection in driving simulation using extreme learning machine. Neurocomputing.

[216] Kumar P., Perrollaz M., Lefevre S., Laugier C. Learning-based approach for online lane change intention prediction. Proceedings of the 2013 IEEE Intelligent Vehicles Symposium (IV).

[217] Beggiato M., Pech T., Leonhardt V., Lindner P., Wanielik G., Bullinger-Hoffmann A., Krems J. (2018). Lane Change Prediction: From Driver Characteristics, Manoeuvre Types and Glance Behaviour to a Real-Time Prediction Algorithm. UR: BAN Human Factors in Traffic.

[218] Krumm J. A Markov Model for Driver Turn Prediction. https://www.microsoft.com/en-us/research/publication/markov-model-driver-turn-prediction/.

[219] Li X., Wang W., Roetting M. (2019). Estimating Driver’s Lane-Change Intent Considering Driving Style and Contextual Traffic. IEEE Trans. Intell. Transp. Syst..

[220] Husen M.N., Lee S., Khan M.Q. Syntactic pattern recognition of car driving behavior detection. Proceedings of the 11th International Conference on Ubiquitous Information Management and Communication.

[221] Kuge N., Yamamura T., Shimoyama O., Liu A. (2000). A Driver Behavior Recognition Method Based on a Driver Model Framework.

[222] Doshi A., Trivedi M.M. (2009). On the roles of eye gaze and head dynamics in predicting driver’s intent to change lanes. IEEE Trans. Intell. Transp. Syst..

[223] McCall J.C., Trivedi M.M. (2007). Driver behavior and situation aware brake assistance for intelligent vehicles. Proc. IEEE.

[224] Cheng S.Y., Trivedi M.M. (2006). Turn-intent analysis using body pose for intelligent driver assistance. IEEE Pervasive Comput..

[225] Li G., Li S.E., Cheng B., Green P. (2017). Estimation of driving style in naturalistic highway traffic using maneuver transition probabilities. Transp. Res. Part C Emerg. Technol..

[226] Bergasa L.M., Nuevo J., Sotelo M.A., Barea R., Lopez M.E. (2006). Real-time system for monitoring driver vigilance. IEEE Trans. Intell. Transp. Syst..

[227] Smith P., Shah M., Lobo N.D.V. (2003). Determining driver visual attention with one camera. IEEE Trans. Intell. Transp. Syst..

[228] Sigari M.-H., Fathy M., Soryani M. (2013). A Driver Face Monitoring System for Fatigue and Distraction Detection. Int. J. Veh. Technol..

[229] Flores M., Armingol J., de la Escalera A. (2010). Driver Drowsiness Warning System Using Visual Information for Both Diurnal and Nocturnal Illumination Conditions. EURASIP J. Adv. Signal Process..

[230] Wang R.-B., Guo K.-Y., Shi S.-M., Chu J.-W. A monitoring method of driver fatigue behavior based on machine vision. Proceedings of the IEEE IV2003 Intelligent Vehicles Symposium, Proceedings (Cat. No.03TH8683).

[231] Zhang Z., Zhang J.S. Driver Fatigue Detection Based Intelligent Vehicle Control. Proceedings of the 18th International Conference on Pattern Recognition (ICPR’06).

[232] Wenhui D., Xiaojuan W. Fatigue detection based on the distance of eyelid. Proceedings of the 2005 IEEE International Workshop on VLSI Design and Video Technology.

[233] Lalonde M., Byrns D., Gagnon L., Teasdale N., Laurendeau D. Real-time eye blink detection with GPU-based SIFT tracking. Proceedings of the Fourth Canadian Conference on Computer and Robot Vision (CRV ‘07).

[234] Batista J. A Drowsiness and Point of Attention Monitoring System for Driver Vigilance. Proceedings of the 2007 IEEE Intelligent Transportation Systems Conference.

[235] Audi|Luxury Sedans, SUVs, Convertibles, Electric Vehicles & More. https://www.audiusa.com.

[236] Bayerische Motoren Werke AG The International BMW Website|BMW.com. https://www.bmw.com/en/index.html.

[237] National Highway Traffic Safety Administration (2010). Crash Factors in Intersection-Related Crashes: An on-Scene Perspective. Nat. Center Stat. Anal..

[238] Ford Ford–New Cars, Trucks, SUVs, Crossovers & Hybrids|Vehicles Built Just for You|Ford.com. https://www.ford.com/.

[239] Mercedes-Benz International News, Pictures, Videos & Livestreams. https://www.mercedes-benz.com/content/com/en.

[240] New Cars, Trucks, SUVs & Hybrids|Toyota Official Site. https://www.toyota.com.

[241] National Highway Traffic Safety Administration (2016). Federal Automated Vehicles Policy: Accelerating the Next Revolution in Roadway Safety.

[242] Lee J.D. (2014). Dynamics of Driver Distraction: The process of engaging and disengaging. Ann. Adv. Automot. Med..

[243] Fridman L., Brown D.E., Glazer M., Angell W., Dodd S., Jenik B., Terwilliger J., Patsekin A., Kindelsberger J., Ding L. (2019). MIT advanced vehicle technology study: Large-scale naturalistic driving study of driver behavior and interaction with automation. IEEE Access.

[244] Su D., Li Y., Chen H. (2019). Toward Precise Gaze Estimation for Mobile Head-Mounted Gaze Tracking Systems. IEEE Trans. Ind. Inform..

